# CD24 expression does not affect dopamine neuronal survival in a mouse model of Parkinson's disease

**DOI:** 10.1371/journal.pone.0171748

**Published:** 2017-02-09

**Authors:** Simon R. W Stott, Shaista Hayat, Tom Carnwath, Shaady Garas, Jonathan P. Sleeman, Roger A. Barker

**Affiliations:** 1 John van Geest Centre for Brain Repair, E.D. Adrian Building, Forvie Site, Robinson Way, Cambridge, England; 2 Medical Faculty Mannheim, University of Heidelberg, Mannheim, Germany; 3 Institute of Toxicology and Genetics, Karlsruhe Institute of Technology (KIT), Karlsruhe, Germany; 4 Wellcome Trust-MRC Stem Cell Institute, Cambridge, England; Thomas Jefferson University, UNITED STATES

## Abstract

Parkinson’s disease (PD) is a progressive neurodegenerative condition that is characterised by the loss of specific populations of neurons in the brain. The mechanisms underlying this selective cell death are unknown but by using laser capture microdissection, the glycoprotein, CD24 has been identified as a potential marker of the populations of cells that are affected in PD. Using in situ hybridization and immunohistochemistry on sections of mouse brain, we confirmed that CD24 is robustly expressed by many of these subsets of cells. To determine if CD24 may have a functional role in PD, we modelled the dopamine cell loss of PD in *Cd24* mutant mice using striatal delivery of the neurotoxin 6-OHDA. We found that *Cd24* mutant mice have an anatomically normal dopamine system and that this glycoprotein does not modulate the lesion effects of 6-OHDA delivered into the striatum. We then undertook in situ hybridization studies on sections of human brain and found—as in the mouse brain—that CD24 is expressed by many of the subsets of the cells that are vulnerable in PD, but not those of the midbrain dopamine system. Finally, we sought to determine if CD24 is required for the neuroprotective effect of Glial cell-derived neurotrophic factor (GDNF) on the dopaminergic nigrostriatal pathway. Our results indicate that in the absence of CD24, there is a reduction in the protective effects of GDNF on the dopaminergic fibres in the striatum, but no difference in the survival of the cell bodies in the midbrain. While we found no obvious role for CD24 in the normal development and maintenance of the dopaminergic nigrostriatal system in mice, it may have a role in mediating the neuroprotective aspects of GDNF in this system.

## Introduction

The primary clinical features of Parkinson’s disease (PD)—classically defined as a resting tremor, bradykinesia, postural instability and rigidity [1]—are associated with a >60% loss of dopamine (DA) neurons in the substantia nigra pars compacta (SNpc) of the midbrain [2]. The severe cell loss observed in the SNpc is in contrast, however, with that seen in the adjacent midbrain DA populations: the ventral tegmental area (VTA) which has only a 40% reduction in DA cells [2,3] and the retrorubral field (RRF) which exhibits little or no DA cell loss [2,4]. This raises questions as to why the SNpc population of DA neurons is more vulnerable to the PD disease process.

This selective cell loss is though not restricted to the midbrain DA populations. Post-mortem analysis of brains from early stage PD patients has suggested that the loss of the DA neurons in the SNpc is preceded by cell death in specific nuclei further down the brainstem, in particular the dorsal motor nucleus of the vagal nerve (DmnV), the raphe nucleus (RN), the pedunculopontine nuclei (PPN), and the locus coeruleus (LC) [5]. Remarkably, the nuclei neighbouring these structures remain significantly less affected, again raising questions regarding the selective cell loss associated with PD.

Given all this, one can hypothesize that the susceptible cells may share certain common features that makes them vulnerable to the disease, and one such factor that we identified was CD24 (cluster of differentiation 24; also known as Cd24a, M1/69-J11D heat-stable antigen (HSA), Lymphocyte antigen 52 (Ly-52), or Nectadrin). CD24 is a glycosyl phosphatidylinositol (GPI) anchored sialoglycoprotein that is expressed in a variety of tissues [6], and when first cloned, was associated with T-cell co-stimulation [7]. In lymphoid organs, this feature of *Cd24* is redundant, only becoming essential in the absence of Cd28 [8,9]. In “co-stimulator ligand rich” organs, such as the central nervous system (CNS), however, the role of *Cd24* is more critical. *Cd24* deficiency in mouse models of experimental autoimmune encephalomyelitis (EAE) prevents T-cell proliferation and persistence [10], while conditionally over-expressing the gene exaggerates the response [11]. It has also been reported that, through an interaction with SiglecG (in mice) or Siglec10 (in humans), CD24 selectively represses the host response to injury, discriminating danger-associated molecular patterns (DAMPs) from pathogen-associated molecular patterns (PAMPs) [12].

Laser capture microdissection gene expression studies have demonstrated that *Cd24* is expressed in the SNpc, but not the VTA [13–15] as well as the DmnV [16] and the LC [17]. Given the shared expression of *Cd24* amongst the nuclei specifically affected in PD, and the direct interaction of *Cd24* with the immune system, we sought to determine whether this protein could have a role in the cell loss seen in PD.

We found that CD24 is expressed in both the mouse and human brain in many of the nuclei affected pathologically in PD, but curiously not the SNpc in the human brain. Furthermore, we found using a classical mouse model of PD that while the absence of *Cd24* did not impact on the long-term survival of the DA system, it did affect the neuroprotective effects of Glial cell-derived neurotrophic factor (GDNF) at the level of the striatal dopaminergic fibres. This result may be particularly relevant given the absence of CD24 in the SNpc neurons in the human adult midbrain.

## Materials and methods

### Mice and post-mortem human tissue

*Cd24* knockout mice bred on a C57/Black background were used in this study [18]. The wild-type (*Cd24+/+*) and homozygote (*Cd24*-/-) littermates were housed with unrestricted access to food and water. All animal procedures were performed in accordance with Cambridge University animal care committee regulations. Experiments were performed under the Home Office licences PPL 80/2366 (expired April 2016) or 70/8411 (valid until 2020).

Samples of human tissue were obtained from the Parkinson’s UK Tissue Bank, funded by Parkinson's UK, a charity registered in England and Wales (258197) and in Scotland (SC037554), and from the Cambridge Brain Bank at Addenbrooke's Hospital (Cambridge) which is supported by the NIHR Cambridge Biomedical Research Centre. Handling of human tissue was done according to the UK Human Tissue Act 2006. Ethical approval for the research conducted in this current study was granted by the Cambridge Central Ethics (ref no 01/177).

### Behavioural testing

#### Cylinder test

The cylinder test was conducted as previously described with rats [19,20]. Briefly, the mice were placed in a glass cylinder (13cm wide; 18 cm high) with two vertical mirrors positioned behind it, allowing for the paws of each mouse to be visualised at all times. Recordings were made with a Logitech c170 webcam, commencing as soon as the mouse was placed in the cylinder, and were analysed by an experimentally blind investigator (before genotyping). Six recordings lasting 3 minutes each were made over the last nine days before the mice were sacrificed. The first 20 wall contacts were counted in each session, and the frequency of ipsilateral (same side as the lesion) versus contralateral paw contacts was determined. Full extension of the digits of each paw was required for the contact to be counted.

#### Amphetamine induced rotations

Amphetamine induced rotations were induced on the day before the mice were to be sacrificed. The mice were injected intraperitoneally with 2.5mg/kg of amphetamine and placed in glass cylinders (13cm wide; 18 cm high). The number of rotations over 50 minutes was recorded on a Logitech c170 webcam and counted by an experimentally blind investigator. The number of full rotations was recorded for 1 minute every 5 minutes, and expressed as the net number of ipsilateral turns/min.

#### Open field locomotion test

The open field locomotion measure was conducted on the day before sacrifice. It involved a 10-minute recording session in which the mice were placed in an arena (70cm x 55cm x 22cm), with a Logitech c170 webcam recording the movement of the mice from above the enclosure. Footage of the test session was analysed using Icy "Mice Profiler Tracking" software (http://icy.bioimageanalysis.org/) [21,22].

### Stereotaxic injections

The mice were anesthetized with isoflurane, placed in a stereotaxic frame, and were unilaterally lesioned in the striatum (co-ordinates: AP: +1.5; ML: +0.21; DV: 3.0). A small hole was drilled in the exposed skull and a Hamilton syringe was used for the delivery of the 6-OHDA (5μg/μl; 1.5μl in total, at a rate of 0.5μl per minute). The syringe was left in place for 5 minutes before being slowly removed. The mice were then sutured and allowed to recover until either 12 or 70 days post-surgery. Special husbandry was provided post-surgical procedures, with mice being weighed daily and provided with subcutaneous injections of warm saline (at least 0.3ml/day), mashed pellets, and chocolate balls (Supreme Mini Treats from Datesand) to prevent weight loss.

### Tissue processing

#### Mouse

The mice used in this study were euthanized with a 0.3 ml intraperitoneal injection of Euthatal (pentobarbitone sodium, 200 mg/ml; Merial, UK). They were then perfused transcardially with either 0.9% saline followed by ice-cold 4% paraformaldehyde (0.1 M phosphate buffer, pH 7.4) or formalin solution (10%; Sigma Aldrich). Brains were removed and post-fixed in their respective solutions overnight. The PFA fixed brains were then shifted to 30% sucrose, while the formalin fixed brains were washed in PBS and switched to 70% ethanol for paraffin embedding.

#### Human

Post-mortem tissues obtained from both the Parkinson's UK brain bank at Imperial College (London) and the Addenbrooke's Brain Bank, were sectioned at source and provided as either fresh frozen sections (12μm thick) or paraffin embedded sections (6μm).

### Immunohistochemistry

Perfused brains were sectioned coronally at 35 μm intervals using a sledge microtome (Leica SM 1400) and collected in 6 series in antifreeze-based solution. Immunohistochemistry was performed as previously described [23]. Briefly, free-floating sections were washed in 0.1M phosphate buffer saline (PBS) before being quenched for 20–30 min in 3% H_2_O_2_ and 50% methanol. The sections were then washed in PBS again, before being incubated overnight in 5% normal horse serum plus 0.05% Triton X-100 in PBS at room temperature with a primary antibody recognizing Rat-CD24 (M1/69; sc-19651, 1:50; Santa Cruz), rat-dopamine transporter (DAT; 1:200; MAB369, Chemicon), Goat-GDNF (1:500; AF-212-NA; R&D systems), and Rabbit-TH (1:1000, Pelfreez). A one-hour incubation with an appropriate biotinylated secondary antibody (1:200; Vector Laboratories, Burlingame, CA) was followed by an additional 40-minute incubation in avidin-biotin-peroxidase solution (ABC Elite; Vector Laboratories). Finally, visualization of the bound antibody was done using 3'-diaminobenzidine (DAB; Sigma). The sections were then mounted on gelatin-coated glass slides, dehydrated in escalating ethanol concentrations, cleared in xylene, and cover slipped with DEPEX Mounting Medium.

Immunofluorescence was performed using the antibodies mentioned above, in addition to other commercially available antibodies: Rabbit-ALDH1A1 (1:200; ab24343, Abcam), Rat-CD11b (1:500; RnD systems), Rat-CD24 (1:50; Santa Cruz), Rabbit-GFAP (1:500; DAKO), Mouse-GFAP (1:500; Abcam), rabbit anti-Girk2 (1:100, APC-006, Alomone Labs), Rabbit-TH (1:1000, Pelfreez), Mouse-TH (1:500, Millipore), and Sheep-TH (1:1000, Millipore). Sections were incubated overnight with combinations of primary antibodies in 5% normal donkey serum plus 0.05% Triton X-100 in PBS at room temperature. Expression was visualised the next day with fluorescent secondary antibodies (Alexafluror 488, 568, & 688; 1:500, Invitrogen). The sections were mounted on gelatin-coated slides and cover slipped with Flurosave (Millipore).

### In situ hybridization

In situ hybridization analysis was conducted on sections of mouse and human brain using the RNAscope Target Probes (Advanced Cell Diagnostics) according to the manufacturer's instructions. For mouse *Cd24*, the probes targeted the 273–1419 base pair region of the gene (Accession No. NM_009846.2), and for human CD24, the probes targeted the 458–2142 base pair region of the gene (Accession No: NM_013230.2).

### Stereological estimations

To quantify the number of TH+ cells in the substantia nigra randomised counts were performed using a standard stereological method (Olympus CAST Grid System) [24]. This analysis was conducted as previously described [23]. Briefly, the substantia nigra was defined by a ventral border passing from the lateral extremity of TH+ cells, down along the dorsal border of the cerebral peduncle to the medial tip of this structure. A vertical line was passed through the medial tip of the cerebral peduncle and represented the medial border of the substantia nigra. The dorsal border of the area of interest extended dorso-laterally along the upper limits of the TH+ cell bodies in the SNpc. On sections where the medial lemniscus was present, TH+ cells superior to this structure were not counted—thus excluding the TH+ cells of the VTA. For all brains, five sections of substantia nigra (at approximately 200μm intervals, between −2.5 and −3.8 relative to Bregma) were counted using a 20x lens. The intact/unlesioned side of the brain was counted with a grid size that was 180x180μm, with the counting frame measuring 80x80μm, while the lesioned side of the brain required a grid size that was 150x150μm, with the counting frame measuring 100x100μm. At least 200 cells were counted per side of the brain and estimates of the total number of neurons were calculated according to the optical fractionator formula, and only a coefficient of error of <0.10 was accepted [25].

### Optical density analysis

All of the DAB stained slides in this study were imaged using the scanscope XT slide scanner (Aperio), at a resolution of 0.5 microns per pixel, using a x20 objective. To quantify the fibre density in the striatum and Substantia nigra pars reticulata (SNpr), the mean optical intensity was measured from the TH-positive stained sections [26,27]. For the striatum, the measurements were conducted on 12 coronal levels at intervals of approximately 210μm, corresponding to sections of the structure from between +1.7 and -1.4 relative to bregma. The area of interest did not include the nucleus accumbens and globus pallidus. Fibre density analysis on TH+ fibres of the SNpr was conducted on only 2 coronal sections per brain (210 μm apart; from approximately -2.7 to -3.2 according to Bregma) in the rostral SNpc as only TH+ fibres (and no TH+ cell bodies) are present there. Nonspecific background was determined by taking measurements from the TH-negative corpus callosum and normalizing it to the white light background surrounding the section on the glass slide. This analysis was conducted using ImageJ (version 1.42q for Mac OSX, from the National Institutes of Health; http://rsb.info.nih.gov/ij/).

### Statistical analysis

All statistical analyses were performed using Prism (version 5.0; Graphpad Software, San Diego, CA, USA). The number of animals and statistical tests used in each experiment is provided in the Results section and in each figure legend (Mann-Whitney (M-W) or ANOVA). For optical density experiments, comparisons were made between the lesioned and intact side of the brain to avoid between-section differences in antibody staining. For all of the stereological estimations of TH+ cells in the midbrain, the lesioned side of the brain was compared with the intact side. Differences were considered significant when P < 0.05 (*P < 0.05; **P < 0.005; ***P < 0.0005). Percentages are presented with the standard error (SE) in both the text and figures.

## Results

### The expression of Cd24 transcripts in the adult mouse brain

**Fig 1:**
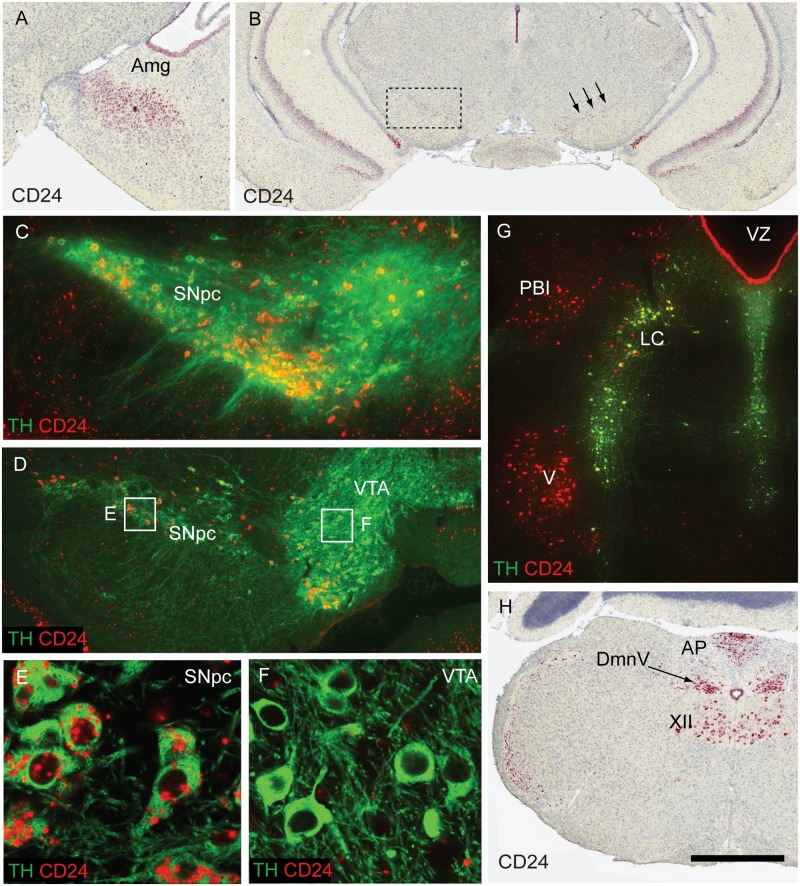
In situ hybridization of *Cd24* expression on the adult mouse brain. (A) A coronal image of *Cd24* expression (in red) in the central amygdala nucleus (Amg). (B) *Cd24* transcripts labelling the Substantia nigra pars compacta (SNpc; arrows) in the midbrain. The dashed line boxed area in B corresponds to the panel in C. (C) *Cd24* expression in the SNpc, overlaid with tyrosine hydroxylase (TH) immunofluorescent staining. (D) A more caudal section of the SNpc to panel C, demonstrating the lack of *Cd24* expression in the ventral tegmental area (VTA). (E) High magnification image of the dashed line boxed area indicated in panel D illustrating the dopamine neuron-specific expression of *Cd24* in the SNpc. (F) A similar image to that of panel E (of the dashed line boxed area indicated in panel D), demonstrating the lack of *Cd24* expression in the dopamine neurons of the VTA. (G) In the hindbrain, *Cd24* transcripts overlapped with the TH+ neurons of the locus coeruleus (LC) and also labelled the parabrachial nucleus (PBl) and the motor nucleus of trigeminal nerve (v). (H) Further down the brainstem, in the medulla, *Cd24* expression was present in the dorsal motor nucleus of the vagal nerve (DmnV), the area postrema (AP), and the hypoglossal nucleus (XII). The scale bar in panel H represents 200μm in panel A&H, 500μm in panel B, 100μm in panels C, D and G, and 50μm in panels E and F.

Using in situ hybridization, we examined *Cd24* transcript expression in the adult mouse brain, confirming and extending those that have been previously reported (Allen Institute for Brain Science. Allen Mouse Brain Atlas [Internet]. Available from: http://mouse.brain-map.org/experiment/show/79591541). Here we shall focus exclusively on those nuclei that are majorly affected in PD, but a full description of *Cd24* expression in the adult mouse brain can be found in S1 Fig. When considering the nuclei preferentially affected in PD [28], we find that *Cd24* expression in the adult mouse brain overlaps with many of these structures. In the forebrain, robust *Cd24* transcript expression is present in the olfactory bulbs (S1A and S1B Fig), though not the anterior olfactory nucleus [29]. We also found strong expression in the amygdala (Fig 1A) [30], but not in the nucleus basalis of Meynert although it was present at high levels in the reticular nucleus of the thalamus (S1I Fig).

In the mesencephalon, *Cd24* transcripts are present in the DA neurons of the SNpc (Fig 1B), as confirmed using double immunofluorescent staining for tyrosine hydroxylase (TH—the rate limiting enzyme involved in DA production; Fig 1C). Quantifications of TH+ cell bodies with more than two red separate aggregations of signal amplification inside the cytoplasm indicated that 60.8%±3.1 of the cell bodies in the SNpc are TH+/*Cd24*+, 31.2%±3.7 are TH+/*Cd24-* and 8.0%±1.2 are TH-/*Cd24+* (n = 3 brains). Double staining with astrocyte (GFAP) and microglial (CD11b) markers found no/little co-localisation in the SNpc and VTA regions (data not shown). *Cd24* expression in the SNpc is more robust in the rostral portion than more caudal aspects (compare Fig 1D and 1C), and in agreement with others we found very little expression of *Cd24* in the VTA when compared to the SNpc [14] (Fig 1E and 1F). This suggests that *Cd24* is a gene that is preferentially expressed by the SNpc DA neurons, which are classically recognised as one of the most affected subsets of cells in the PD brain [2,28].

In the hindbrain, we detected strong labelling of *Cd24* in the LC and parabrachial nucleus (PBl; Fig 1G) [31], and sparse expression in the dorsal raphe nuclei (RN) and Pedunculopontine nuclei (PPN; S1K Fig) [32] as well as the DmnV (Fig 1H). While numerous nuclei in the adult mouse brain express *Cd24*, we considered it noteworthy that many of them (such as the amygdala, SNpc, RN, PPN, PBl, LC, and DmnV) are sites of major pathology in PD [28], while others low in expression (e.g. VTA) are relatively well preserved in this condition.

### CD24 protein expression in the mouse brain

**Fig 2:**
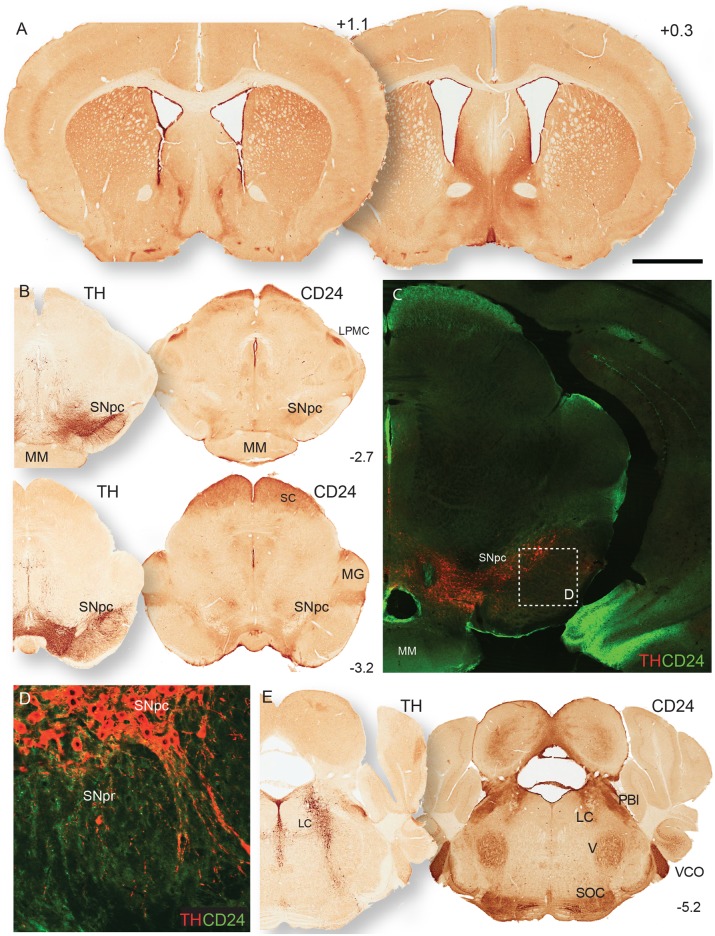
CD24 protein expression pattern in the adult mouse brain. (A) CD24 expression in the mouse forebrain at two levels of the striatum. (B) Coronal sections of the midbrain at the level of the caudal end of the mammillary bodies (MM). TH staining on the left section indicates the location of the dopamine neurons of the SNpc. CD24 labels DA neurons in the SNpc, as well as the lateral posterior thalamic nucleus (LPMC), superior colliculus (SC), and nuclei surrounding the medial geniculate (MG). (C) Immunofluorescent staining of TH and CD24 indicates some localisation in the SNpc. Dashed line box highlighting area of the high magnification image in panel D. (D) CD24 staining on TH+ DA neurons in the SNpc, labelling dendritic fibres projecting down into the substantia nigra pars reticulata (SNpr). (E) TH and CD24 staining in the hindbrain where the LC is labelled by both. Co-ordinates in the right hand corners of panels A, B & E indicate the location of the coronal plane relative to bregma. The scale bar in panel A represents 800μm in panel A&E, 500μm in panel B, 200μm in panel C, and 50μm in panel D.

We followed up the in situ analysis by looking at the protein expression of CD24 in the adult mouse brain. Again, we have focused on the nuclei affected in PD, although a comprehensive representation of CD24 protein localisation is provided in S2 Fig. As previously reported [33], robust CD24 expression was observed in the olfactory bulb (especially the glomerular layer and rostral migratory stream; S2A Fig). We also found a dorsal-ventral gradient of CD24 expression in the rostral striatum (Fig 2A), and strong CD24 expression in the amygdala (S2D Fig). In the midbrain, we observed weak CD24 staining labelling in the SNpc (Fig 2B; S2E–S2G Fig) with double immunofluorescent staining showing low expression of CD24 on the surface of the DA cell bodies—particularly the neurites and dendritic fibres, but no staining in the cytoplasm (Fig 2C and 2D), indicative of a membrane bound protein.

In the hindbrain, we observed very little/no CD24 protein in the raphe nucleus (S2H Fig), and moderate CD24 expression in the cell bodies of the LC (Fig 2E). In the medulla, we found CD24 protein in the DmnV (data not shown). Importantly, while again highlighting an expression profile that strongly overlaps with the sites of PD pathology, the presence of CD24 on the neurites of the DA neurons in the SNpc led us to investigate further the possible function of CD24 in these cells.

### The dopamine system of the Cd24 mutant mouse is anatomically normal

**Fig 3:**
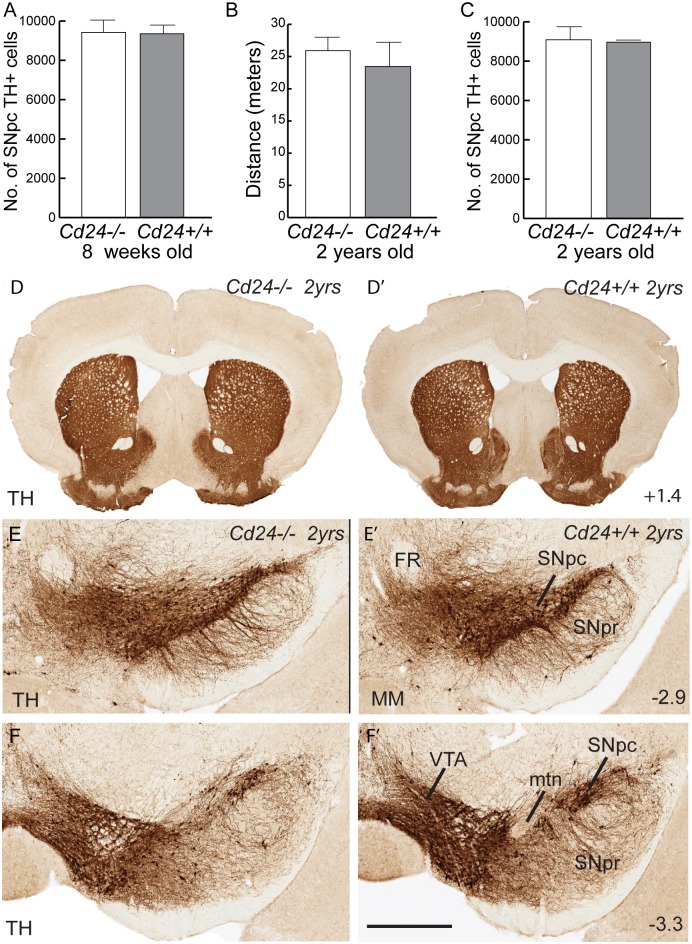
The midbrain dopamine system of the *Cd24* mutant mouse is normal. (A) Stereological estimations of the number of SNpc TH+ cells at 8 weeks of age. (B) Distance travelled in the open field arena by 2-year-old *Cd24-/-* and *Cd24+/+* mice. (C) Stereological estimations of the number of SNpc TH+ cells at 2 years of age. (D) Tyrosine hydroxylase (TH) immunohistochemistry on coronal sections of a brain of a 2-year-old *Cd24-/-* mouse (left column) and a *Cd24+/+* littermate (right column), at the level of the striatum. (E) A coronal section of the SNpc at the level of the fasciculus retroflexus (FR) and the caudal end of the mammillary bodies (MM). (F) Images of the VTA and SNpc at the level of the medial terminal nucleus of the accessory optic tract (mtn). Co-ordinates in the right-hand corners of panels D, E and F indicate location of the coronal plane relative to bregma. The scale bar in panel F represents 1cm in panel D, 200μm in panel E, and 300μm in panel F.

To determine whether *Cd24* has a role in the development or maintenance of the midbrain DA neurons, we analysed the brains of mice that had a targeted deletion of the gene [18]. Immunohistochemistry for CD24 on sections of brain tissue derived from 8week old *Cd24*-/- mice exhibited no labelling, demonstrating that CD24 protein was not present in the brains of these mice (S2I–S2L Fig). Further, TH staining on those same sections indicated no observable difference in the DA system between *Cd24*-/- mice and *Cd24+/+* littermates. In the olfactory bulb, the dopaminergic interneurons in the glomerular layer were in the correct location. The TH+ fibre innervation of the striatum appeared unaltered by the mutation, and stereological estimations of TH+ cells in the SNpc indicated that the absence of *Cd24* did not affect the number of DA neurons in this subregion (Fig 3A; 9422.53±548.3 TH+ cells in the *Cd24*-/- SNpc compared with 9353.2±489.3 in the *Cd24+/+*mice; M-W U = 5, p = 0.86; n = 5 animals per group).

To determine whether the absence of *Cd24* resulted in increased vulnerability for DA neurons with aging, we assessed the *Cd24*-/- mice at 2 years of age. These aged mice displayed no difference in the distance travelled in an open field measure of locomotion (Fig 3B; 25.93±2.69 meters of distance covered by *Cd24*-/- mice compared to 23.46±4.27 for the *Cd24+/+* group; M-W U = 10, p = 0.98; n = 4 animals per group) nor in the number of TH+ neurons in the SNpc (Fig 3C; 8962.71±89.8 TH+ cells in the *Cd24+/+*SNpc compared with 9087.19±566.2 in the *Cd24*-/- mice; M-W U = 4, p = 0.9; n = 4 animals per group). There was no obvious anatomical differences in the dopamine system of the 2 year old *Cd24*-/- mice (S3 Fig), including the fibre innervation of the striatum (Fig 3D and 3D’) and the morphology of the TH+ cells in the SNpc of *Cd24*-/- mice (Fig 3E–3F’), when compared to their *Cd24+/+* littermates. These mutant mice also displayed the correct expression patterns for SNpc-related genes such as G protein-activated inward rectifier potassium channel 2 (Girk2), and aldehyde dehydrogenase 1 family, member A1 (Aldh1a1; data not shown) [34–36]. Together these findings suggested that *Cd24* does not play a critical role in the normal anatomical development and maintenance of the murine midbrain DA system. Whether it has a more subtle role in the synaptic release of DA within the striatum was not explored in this study, but may be worth investigating given the putative role of this molecule at the neuromuscular junction [37].

### CD24 has no effect on striatal 6-OHDA lesions in mouse models of PD

Several reports have highlighted the importance of T-cells in the neurodegeneration associated with neurotoxic and other relevant animal models of PD [38,39]. Given that *Cd24* deficient mice exhibit reduced T-cell proliferation and persistence in models of EAE [10] and conditionally over-expressing the gene exaggerates the phenotype [11], we hypothesized that deletion of the gene may lessen the effect of the neurotoxin 6-OHDA on the long term survival of midbrain DA neurons. Blocking CD24 with antibodies in mice has been shown to not be possible due to toxicity issues ([40] and Prof Peter Altevogt, personal communication), and so we modelled PD in the *Cd24-/-* mouse using striatal delivery of 6-OHDA.

The delivery of the neurotoxin 6-OHDA to the midbrain nigrostriatal pathway is commonly used to model the DA pathology of PD. Injecting 6-OHDA into the medial forebrain bundle or the SNpc results in a very rapid loss of TH+ cells [41,42], which does not allow for a thorough analysis of the molecular events involved in the loss of cells within the midbrain. To circumvent this, we have previously demonstrated that 6-OHDA delivery to the striatum results in a slower rate of midbrain TH+ cell loss, beginning between 9–12 days post lesion, coinciding with local glial activation [23]. Thus, we chose to adopt this approach given also the possible role of *Cd24* in modulating neuroinflammatory processes to injury. This also explains why we examined the brains of the *Cd24-/-* and *Cd24+/+* mice over this period of cell loss following surgery.

**Fig 4:**
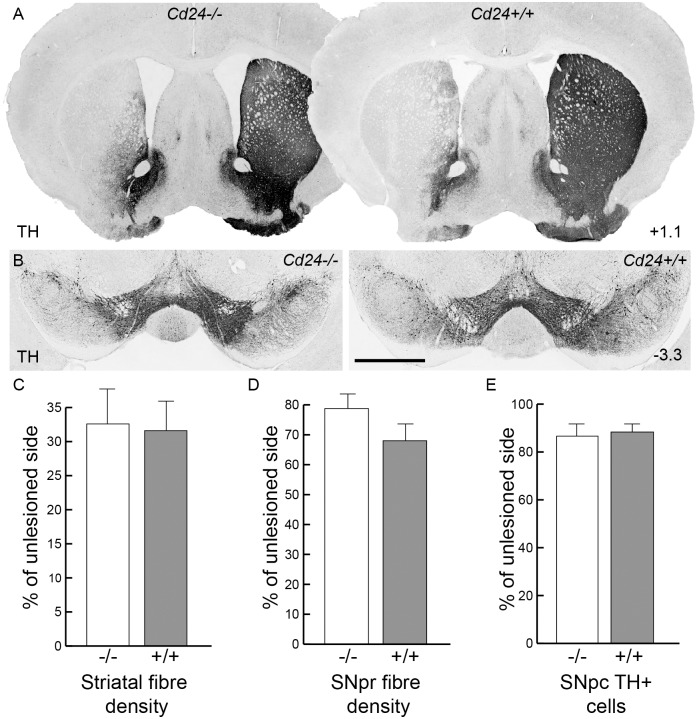
The absence of *Cd24* has no effect on dopaminergic neuronal survival or fibre innervations in the striatal 6-OHDA lesioned mouse model of Parkinson's disease. (A) Tyrosine hydroxylase (TH) immunohistochemistry on coronal sections of brain from a 6-OHDA lesioned *Cd24-/-* mouse (left column) and a *Cd24+/+* littermate (right column) at 12 days post-surgery, at the level of the striatum. (B) Representation sections of midbrain (from the respective brains provided in panel A) illustrating the loss of TH+ cells on the 6-OHDA lesioned side of the brain. (C) Optical density analysis of the TH+ fibres in the striatum indicated no difference between the genotypes. (D) Optical density analysis of the TH+ fibres in the SNpr also showed no significant difference between the genotypes. (E) Stereological estimations of the number of SNpc TH+ cells found no difference between the *Cd24-/-* and *Cd24+/+* mice. Co-ordinates in the right-hand corners of panels A indicate location of the coronal plane relative to bregma. The scale bar in panel A represents 1cm and 500μm in panels B.

At 12 days post-lesion, we found no difference between genotypes in the fibre density of TH+ axonal arborisations in the striatum (33.4%±6.9 of the unlesioned side in the *Cd24-/-*mice compared with 33.3%±7.2 for the *Cd24+/+*mice (n = 5 in both groups); M-W U = 10, p = 0.998; Fig 4A and 4C) or the TH+ dendritic branchings in the SNpr (76.9%±6.3 of the unlesioned side compared with 68.0%±2.5 for the *Cd24+/+* mice; M-W U = 6, p = 0.38; Fig 4B and 4D). We also observed no difference in the number of SNpc TH+ neurons using stereological estimations at 12 days post-surgery (86.6%±4.2 of the unlesioned side for *Cd24*-/- mice compared to 88.3%±2.2 of the unlesioned side for *Cd24+/+* mice; M-W U = 5, p = 0.74; Fig 4E).

**Fig 5:**
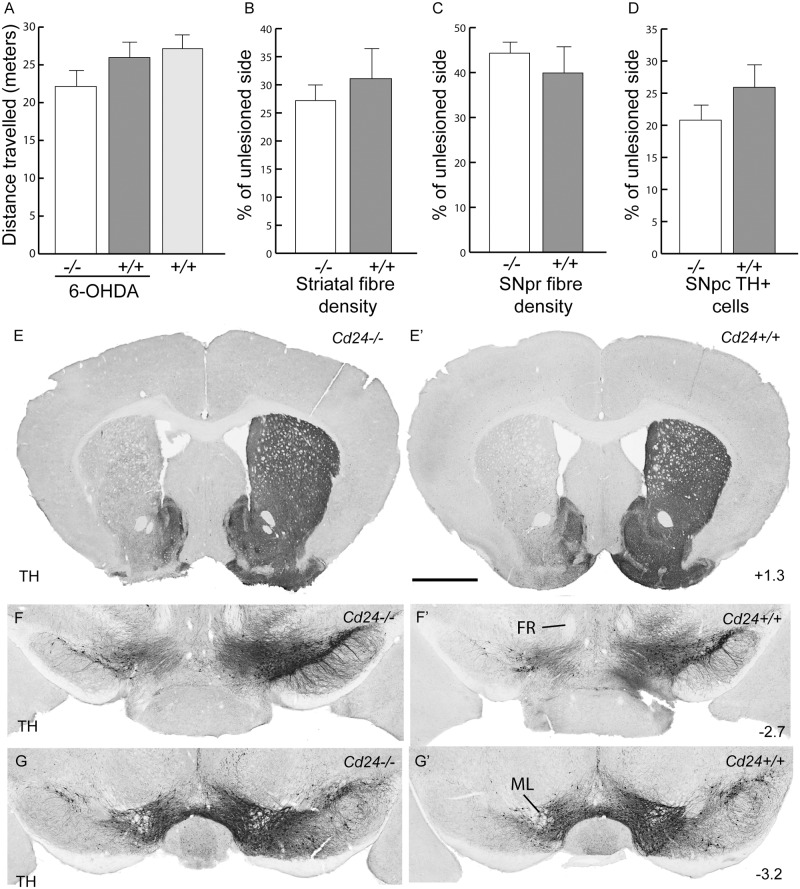
No difference in the long-term structural and functional integrity of the nigrostriatal pathway in the absence of *Cd24* in the 6-OHDA mouse model of Parkinson's disease. (A) Distance travelled in the open field arena by 6-OHDA lesioned *Cd24-/-* and *Cd24+/+* mice at 70 days post-surgery, compared to an unlesioned group of *Cd24+/+* mice. (B) Optical density analysis of the TH+ fibres in the striatum indicated no difference between the genotypes. (C) Optical density analysis of the TH+ fibres in the SNpr also showed no difference between the genotypes. (D) Stereological estimations of the number of SNpc TH+ cells found no difference between the *Cd24-/-* and *Cd24+/+* mice. (E) Coronal sections at the level of the striatum illustrating the unilateral loss of TH+ fibres in the *Cd24-/-* (left) and *Cd24+/+*littermate (right). (F) Coronal sections of the midbrain at the level of the fasciculus retroflexus (FR) demonstrating TH+ cell loss in the rostral SNpc of the *Cd24-/-* (left) and *Cd24+/+* littermate (right). (G) Sections of midbrain at the level of medial lemniscus (ML) intersecting the VTA and SNpc illustrating TH+ cell loss. Co-ordinates in the corners of panels E, F and G indicate location of the coronal plane relative to bregma. The scale bar in panel E represents 1cm in panel E, and 600μm in panels F and G.

To determine whether the absence of CD24 had any long-term consequences on the survival of the DA neurons, we next examined the 6-OHDA-striatal lesioned mice 70 days post-lesion—a time point when the pathological changes linked to the lesion are known to be complete [43]. Before culling the mice, we performed behavioural testing which included the cylinder test, amphetamine induced rotations, and open field locomotion. We found no difference between the mice in any of these tests (*Cylinder test*: affected forelimb usage was 64.3±8.7% of unaffected forelimb in the *Cd24-/-*mice vs 59.3±7.7% in the *Cd24+/+* at 70 days post-6-OHDA lesion, M-W U = 27.5, p = 0.66; compared with 103.8±5.7% in non-lesioned *Cd24+/+* control mice; *Amphetamine induced Rotations*: 4.17±1.01 rotations per minute in the lesioned *Cd24-/-*mice vs 3.45±1.3 in the lesioned *Cd24+/+* mice, M-W U = 11.5, p = 0.33; *Open field test*: 22.15±2.28 meters of distance covered in the lesioned *Cd24*-/- mice vs 25.96±2.8 meters in the lesioned Cd24+/+, M-W U = 9, p = 0.18; compared with 27.14±1.51 meters in non-lesioned *Cd24*+/+ control mice, Fig 5A; n = 8 in each group). Histologically there was no difference in TH+ fibre density in the striatum (27.2±4.1% of unlesioned striatum compared to 31.1±5.8% in the *Cd24+/+* mice; M-W U = 20.5, p = 0.41; Fig 5B, 5E and 5E’; n = 8 in each group) nor in the SNpr (44.3±2.7% of unlesioned SNpr, compared to 39.9±5.5% in the *Cd24+/+* mice; M-W U = 25, p = 0.75; Fig 5C and 5F–5G’). Stereological estimations of the number of TH+ cells in the SNpc showed no significant differences between *Cd24-/-* and *Cd24+/+*mice (20.8±2.5% of unlesioned SNpc, compared to 25.9±3.8% in the *Cd24+/+* mice; M-W U = 15, p = 0.15; Fig 5D and 5F–5G’). Collectively, these results suggest that *Cd24* has no impact on the long-term survival of the SNpc DA neurons following a 6-OHDA striatal lesion.

### CD24 is involved in the neuroprotective effect of GDNF

Given that the absence of *Cd24* had no impact on the overall survival of SNpc DA neurons in the 6-OHDA model of PD, we next sought to determine whether *Cd24* may have more of a role in mediating the neuroprotective effects of GDNF. Christophersen et al [44] reported a nine fold increase in *Cd24* expression in the substantia nigra following lentiviral delivery of GDNF into the striatum. Thus, we reasoned that *Cd24* may be involved in some aspect of the neuroprotective effects of GDNF. To test this hypothesis, we delivered AAV-GDNF into the striatum of both WT and *Cd24-/-*mice.

One-month post-surgery, robust GDNF expression was observed in the striatum of both groups of mice (optical density analysis indicated a 440.4±56.9% increase in GDNF staining in the injected striatum compared to the uninjected side of the *Cd24-/-* mice versus with 428.7±75.7% in the injected *Cd24+/+* mice; M-W U = 12, p = 0.94; S4A Fig). Both sets of mice also exhibited a reduction in body weight in accordance with previous reports [45], suggesting that GDNF functions normally in *Cd24-/-* mice. Increased levels of CD24 were present in the SNpc of the transfected side of the brain in the *Cd24+/+* mice, but absent (as one would expect) in the *Cd24-/-* mice (S4B and S4B’ Fig).

Two months post-AAV-GDNF transfection, both groups of AAV-GDNF injected mice (and a control group of WT mice; n = 5 in each group) received a striatal delivery of 6-OHDA on the same side of the brain. By 21 days post lesion, the loss of TH+ fibres in the striatum has plateaued and remains stable. When we compared the 21 day time point data with that of 70 days post lesion (see above), we found TH+ fibre loss of 39.8%±7.2 of intact side and 38.2%±8.6 for the 21 and 70 day timepoints, respctively (M-W U = 12, p = 0.43; S5A and S5B Fig). A similar stability is also observed in the number of TH+ cell bodies SN (36.6%±2.9 of intact side for 21 days compared to 35.5%±10.1 for 70 days; M-W U = 11, p = 0.34; S5C and S5D Fig), indicating that the 21 days time point can be used to determine the final extent of the lesion in this striatal 6-OHDA model.

**Fig 6:**
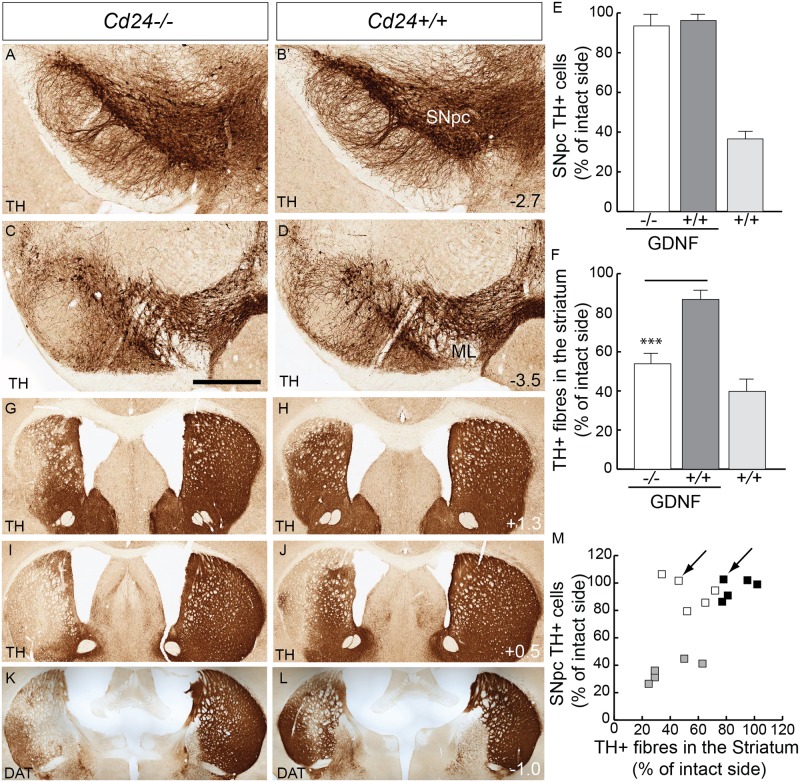
CD24 is important for the neuroprotective effect of GDNF on dopaminergic fibres, but not cell bodies, in the 6-OHDA striatal lesion model of PD. (A-D) Tyrosine hydroxylase (TH) immunohistochemistry on coronal sections of a midbrain from AAV-GDNF transfected—6-OHDA lesioned *Cd24-/-* mouse (left column) and a *Cd24+/+* littermate (right column) at 12 days post-surgery, at the level of the rostral SNpc. (A&B) and caudal SNpc (C&D). (E) Stereological estimations of the number of SNpc TH+ cells found no difference with AAV-GDNF treatment in the 6-OHDA lesioned *Cd24-/-* and *Cd24+/+* mice, but there is a severe reduction in the 6-OHDA only lesioned *Cd24+/+ mice* (controls). (F) Optical density analysis of the TH+ fibres in the striatum indicated a significant difference between the AAV-GDNF/6-OHDA lesioned *Cd24-/-* and AAV-GDNF/6-OHDA *Cd24+/+* mice. (G-L) TH staining of fibre loss in the rostral (G and H), medial (I and J) and caudal (K and L) striatum from an AAV-GDNF/6-OHDA lesioned *Cd24-/-* mouse (left column) and a AAV-GDNF/6-OHDA *Cd24+/+* littermate (right column). Panels K&L were stained with DAT, indicating that the effect was not specific for TH. (M) A dot-plot graph comparing the reduction in TH+ cells in the SNpc with the loss of TH+ fibres in the striatum. Black boxes represent *Cd24+/+* that received both AAV-GDNF and 6-OHDA, white boxes represent *Cd24-/-* that received both AAV-GDNF & 6-OHDA, and grey boxes represent *Cd24+/+* (controls) that received only 6-OHDA. The arrows in panel M indicate the two mice that are presented in panels A-D & G-L. The scale bar in panel D represents 400μm in panels A-D, and 800μm in panels G-L.

At three weeks post-6-OHDA lesion, we found no difference in the reduction of SNpc DA neurons on the lesioned side of the brain when comparing the two groups of AAV-GDNF injected mice (93.5±5.0% of the unlesioned side in the *Cd24-/-* mice compared with 96.2 ±3.2% in the *Cd24+/+* mice; M-W U = 10, p = 0.66; Fig 6A–6E). In the group of WT mice that received striatal delivery of 6-OHDA, but no AAV-GDNF, only 36.6±2.9% of the SNpc TH+ neurons remained compared to the intact side (Fig 6E). These results suggest that CD24 is not involved in the neuroprotective effects of GDNF at the cell body level. However, when we conducted optical density analysis of the TH+ fibres in the striatum, we did observe a significant difference between the two groups of mice that received AAV-GDNF (53.5±6.8% of the intact side in the *Cd24-/-* mice compared with 86.8 ±5.1% in the *Cd24+/+* mice; M-W U = 0, p = 0.0079; Fig 6F–6M; S4C–S4F′ Fig). The same result was also observed with Dopamine Transporter (DAT) staining (Fig 6K and 6L), indicating that the effect was not specific for TH. This result indicates that CD24 plays a role in the GDNF-induced neuroprotection of DA neurons at the level of the striatal neurites, which also fits with our earlier expression data on CD24.

### CD24 expression in the human brain

Finally, in order to compare these mouse results with the human situation, we looked at *CD24* expression in the human brain. Attempts to label *CD24* transcripts on post-mortem brain tissue using conventional in situ hybridization procedures proved very difficult. DIG-labelled in situ probes provided little if any signal above background (including the use of previously published protocols [46]; on both paraffin embedded and fresh frozen tissue from 5 brains; average post-mortem interval = 22hrs; range = 6–47 hours). To resolve this situation, we utilised a DNA-branching protocol (RNAscope, Advanced Cell Diagnostics). Using this approach on fresh frozen, PFA-fixed sections (from 5 normal brains and 5 PD brains supplied by the Parkinson's UK Brain Bank; Table 1), we successfully investigated *CD24* expression across 9 regions of the brain: medulla, pons, midbrain, amygdala, thalamus, subventricular zone (SVZ), hippocampus and frontal cortex.

**Table 1:** List of post-mortem brains used in the current study.

**CONTROL BRAINS**					
**BRAIN**	**AGE**	**SEX**	**PMI**	**DURATION**	**PATHOLOGICAL FEATURES**
CT01	76	F	6	N/A	Normal/unremarkable brain
CT02	67	M	14	N/A	Normal/unremarkable brain
CT03	56	M	22	N/A	Normal (Unexplained hippocampal sclerosis)
CT04	61	F	71	N/A	Normal. Mild loss neurones in the SNpc, but no lewy bodies or pale bodies. Unremarkable brain.
CT05	78	M	77	N/A	Alzheimer’s Braak 3—insufficient for a diagnosis
CT06	82	F	26	N/A	Normal/unremarkable brain
CT07	84	M	48	N/A	Brain oedema; small thalamic infarct
CT08	71	M	52	N/A	Mild hypoxic changes
CT09	77	M	48	N/A	Presence of Marinesco bodies in SNpc
CT10	81	F	15	N/A	Some microvascular chages
CT11	74	F	29	N/A	Normal/unremarkable brain
**PARKINSON’S DISEASE BRAINS**					
**BRAIN**	**AGE**	**SEX**	**PMI**	**DURATION**	**PATHOLOGICAL FEATURES**
PD01	77	M	46	N/A	Parkinson’s disease—Braak 2
PD02	89	F	34	N/A	Parkinson’s disease—Braak 3
PD03	70	F	42	23	Parkinson’s disease—Braak 3
PD04	88	F	53	7	Parkinson’s disease—Braak 4
PD05	78	M	16	10	Parkinson’s disease—Braak 4 + AB path
PD06	80	M	46	14	Parkinson’s disease—Braak 4 + AB path
PD07	84	M	42	10	Parkinson’s disease—Braak 4 + AB path

Control and Parkinson’s disease (PD) brains used in the present study, with their age of death, sex, post-mortem interval (PMI), and pathological features (based on the pathologist’s notes). For the PD brains, the duration of disease is also provided. The brains supplied by the Parkinson’s UK Brain bank are indicated in the grey boxes.

**Fig 7:**
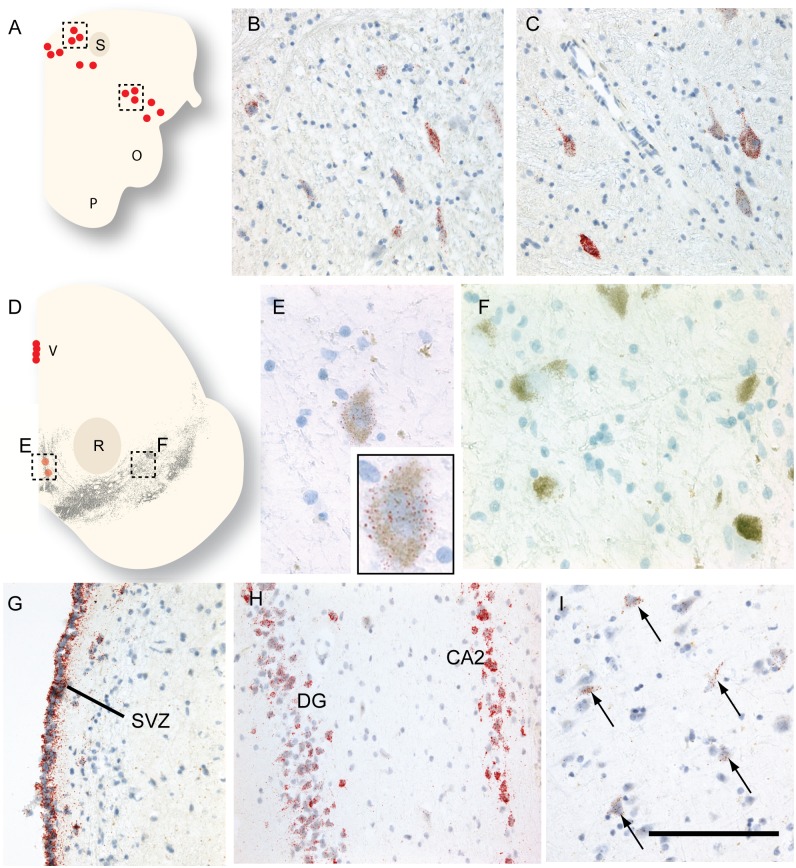
In situ hybridization of *CD24* expression in the adult human brain. (A) A schematic of the medulla oblongata at the level of the pyramid (p), solitary nucleus (s), and the olivary nucleus (o). (B) *CD24* transcripts in the dorsal motor nucleus of the vagal nerve. (C) Neurons expressing *CD24* in the nucleus ambiguus. (D) A schematic of the midbrain at the level of the red nucleus (r) and the neuromelanin+ dopamine neurons (in grey). (E) An individual neuromelanin+ cell close to the midline weakly expressed *CD24* (see inset for higher magnitude). (F) Neuromelanin+ cells in the SNpc, however, are void of *CD24* labelling. (G) The lining of the subventricular zone (svz) at the level of the thalamus presented strong *CD24* expression. (H) *CD24* expression in the dentate gyrus (DG) and CA2 region of the hippocampus. (I) Weakly labelled *CD24*+ cell bodies in the amygdala. The scale bar in panel I represents 150μm in panels B and C, 100μm in panels E and F, and 250μm in panels G-I.

Overall, we found less wide spread expression of *CD24* when compared to the mouse brain. There was also little difference in transcript expression between normal and PD brains, except in cases where reduced cell numbers in particular nuclei resulted in less labelling. In the brain stem, we observed strong *CD24* expression in the large cell bodies of the DmnV (Fig 7A and 7B) and the hypoglossal nucleus of the medulla, with the transcripts being found in the nucleus, cytoplasm and extending into the neurites. *CD24* was also expressed by large cell bodies in the nucleus ambiguous (Fig 7A and 7C) but we were unable to localise the LC on any of the samples of the pons. In the midbrain, the lining of the ventricles robustly expresses *CD24*, but the neuromelanin+ cells of the VTA and SNpc exhibited no *CD24* expression. A very small fraction of the medially-located neuromelanin+ cells per section were very weakly positive for *CD24* (Fig 7D and 7E), but the more laterally positioned SNpc neuromelanin+ cells were completely void of *CD24* transcripts (Fig 7D and 7F). We supplemented the number of midbrain sections with tissue collected from the Cambridge Brain Bank (6 controls and 2 PD; Table 1), but we found only one of the 11 control brains and none of the 7 PD brains exhibited *CD24* expression in the SNpc neuromelanin+ cells (control brain CT03 on Table 1). Although this brain exhibited no disease-related pathology in the midbrain, there was unexplained sclerosis in the hippocampus, indicating the possibility of some additional complication in this case that may have affected the staining.

Similar to the mouse brain, we found very strong *CD24* transcript signal present in all areas of the SVZ examined (Fig 7G). In the hippocampus, we found robust *CD24* transcripts in the dentate gyrus, CA2 and CA3 regions, but not CA1 or CA4 (Fig 7H). The amygdala exhibited weak *CD24* expression (Fig 7I), with transcripts also found in layer 3 of the neighbouring periamygdaloid cortex. In the cortex, we found weak *CD24* expression in layers 1–3, and little or no expression in the lower levels (data not shown). Collectively, these results indicate that *CD24* expression in the human resembles the mouse in many areas of the brain, but importantly it appears to be absent in the neuromelanin+ dopamine neurons of the midbrain.

## Discussion

Understanding the mechanisms involved in the selective cell death associated with PD would represent a major step forward in better understanding and possibly treating this condition. We hypothesized that the nuclei preferentially affected in PD may share a common feature that renders them vulnerable to the causative agent/conditions. Given the specific expression and interactions of CD24 in mice, we considered this gene to be worthy of further investigation.

Our initial expression analysis confirmed and extended previous reports of *Cd24* expression in the rodent brain ([33]; Allen Institute for Brain Science. Allen Mouse Brain Atlas [Internet]. Available from: http://mouse.brain-map.org/experiment/show/79591541) and also provides unique data on the expression of *CD24* in the human CNS. Similarities in expression localisation across species were of interest, especially in evolutionarily older structures such as the brainstem (e.g. the DmnV and the hypoglossal nucleus). The absence of *CD24* expression in the human SNpc, however, was particularly striking, although cross-species differences in gene expression between mice and humans in midbrain DA neurons has been previously reported. Orthodenticle Homeobox 2 (OTX2), for example, is a transcription factor that is expressed by midbrain DA progenitors during development in both mice and humans, and defines the VTA subpopulation in the mouse adult brain, but it is not expressed in any midbrain DA neurons in aged humans [47]. Of the 18 human midbrains analysed in the present study, only one had any *CD24* expression in neuromelanin+ cells, and those cells resided in the more medially located VTA population. As indicated in the results section, that particular case had other pathologies which complicates the interpretation of this finding. Despite this absence of *CD24* in the human adult midbrain DA neurons, we were still interested in assessing possible functions of CD24 in the mouse SNpc DA neurons.

The absence of a phenotype in the midbrain DA system of the *Cd24-/-* mouse—out to 2 years of age—indicates that the gene is not required for the normal anatomical development or maintenance of this population of neurons. While this lack of phenotype was useful for subsequent disease modelling experiments, our analysis could not cover all aspects of DA neuronal function (such as dopamine release, etc). It has been reported that *Cd24* inhibits the extension and collateral branching of neurites through its interactions with neural recognition molecule L1 [48,49], thus in the absence of the gene it could be hypothesized that neurons expressing *Cd24* in the adult brain may exhibit more neurites. Given the density of normal SNpc TH+ axonal arborisations, any increase in branching was difficult to analyse in the *Cd24-/-* mouse and further microscopic analysis may reveal some subtle specific differences. *Cd24* also has a role in synaptic transmission [37]. In the absence of *Cd24*, neuromuscular junctions exhibited synaptic depression, cyclical periods of complete transmission failure, and a reduction in the pool of recycling vesicles in the terminals [37]. Thus, although it was not investigated in the current study, looking at synaptic transmission in the SNpc DA neurons of *Cd24-/-* mice would be of interest.

The modelling of PD (using striatally delivered 6-OHDA) in the *Cd24-/-* mouse showed that the absence of the gene had no impact on the survival of the SNpc DA neurons. However, while CD24 does not exhibit direct neuroprotection, it may be important in mediating the neuroprotective effects of other agents such as GDNF. Thus, we sought to investigate this using AAV-GDNF transfection and 6-OHDA lesions in *Cd24-/-* and wild-type mice and found that there was a greater TH+ fibre density loss in the striatum of the AAV-GDNF/6-OHDA *Cd24-/-* mice. Previously we have demonstrated that the fibre loss following striatal delivery of 6-OHDA in the mouse is very rapid (>60% of eventual fibre loss occurring within the first 24 hours), and that from 3 days post-lesion the extent of fibre loss remains constant [23]. In the current study, while AAV-GDNF preserved the DA cell bodies in the SNpc of the *Cd24-/-* mice, it was not able to rescue the density of striatal TH+ fibres at three weeks post-lesion. The oxidation and washout of 6-OHDA in the brain within the first 24 hours would render a delayed/slowed loss of TH+ fibres unlikely. One confounding aspect of this result, however, is why the absence of CD24 does not result in a similar loss of TH+ fibres to that observed in the 6-OHDA lesioned control mice. This suggests that additional GDNF-related pathways may be involved, resulting in only a partial reduction in the neuroprotective effect of GDNF at the neurite level. This effect, though, does raise the possibility that CD24 may be playing a role in normal DA neurite outgrowth–an area we are now investigating further in vitro.

Many Tgf-β family members are known to up-regulate the expression of CD24. Lentiviral delivery of GDNF into the striatum resulted in an increase in *Cd24* expression in the rodent SNpc [44], and increased *Cd24* promoter activity and *Cd24* mRNA results from forced expression of Artemin [50]. Recently, a reciprocal relationship between CD24 and TGF-β3 in bone marrow-derived stromal cells has been reported [51]. Given the lack of *CD24* expression in the human DA neurons and the reduction in TH+ fibre density in the AAV-GDNF/6-OHDA in the present study, it is tempting to speculate on the role of CD24 and the likely success of clinical trials involving the delivery GDNF to the striatum in people with PD.

As to a specific role CD24 may be playing in PD, it is difficult to speculate. While *CD24* has not been associated with PD, polymorphisms in this gene does result in increased risk and progression of several autoimmune diseases, including multiple sclerosis [52,53], Crohn’s disease [54], and rheumatoid arthritis [55]. This may be relevant to PD given the recent interest in the possibility that PD may have a primary neuroinflammatory component. In addition while there is a lack of *CD24* expression in the DA neurons of the midbrain in humans, the selective expression in other vulnerable regions of the PD brain (particularly in the lower brain stem) suggests that this is a gene worthy of further investigation as it may have some indirect role in the disease process.

In conclusion, we have shown that *CD24* has some commonality of expression with the widespread distribution of pathology seen in PD, although we have been unable to show any causal links between this glycoprotein and the loss of dopamine cells in the mouse model of PD. Nevertheless, we have shown some association between the effects of a known neurotrophic factor for dopaminergic nigral neurons, GDNF, and CD24 expression. This merits further exploration not only as this agent is in clinical trials in patients with PD but also because the benefits of GDNF have been shown to critically depend on other pathways within the striatum [56].

## Supporting information

S1 Fig In situ hybridization of *Cd24* transcript expression on coronal sections of brains from 8-week old adult mice. (A) *Cd24* transcript (red; with haematoxylin counterstain) expression is present in the olfactory bulbs. (B) A high magnification image of the dashed boxed region in panel A, providing *Cd24* expression overlapped with TH immunofluorescent staining in the olfactory bulb. (C) Robust *Cd24* transcripts in the rostral migratory stream (RMS) and 'salt and pepper' distribution throughout the frontal cortex. (D) Strong expression of *Cd24* in the lining of the ventricular zone (VZ) across all levels of the striatum (Str). (E) *Cd24* expression at the level of the thalamus. (F) The dashed-boxed area of panel E, providing a high magnification image of *Cd24* expression in the suprachiasmatic nucleus (SCN). (G) An image of the third ventricle (3V) at the level of the caudal hypothalamus; arrows indicating the area of no *Cd24* expression (a consistent feature across 3 brains analysed). (H) *Cd24* expression in the cortex, layers are indicated on the right-hand side. (I) *Cd24* expression in the hippocampus, especially within the dentate gyrus (DG) and CA1, but not CA3. (J) Immunofluorescent staining of TH expression in the Striatum (Str) and the neighbouring globus pallidus (GP), in combination with *Cd24* expression in the Reticular nucleus of the thalamus (RT) and lining of the VZ. (K) In the hindbrain, *Cd24* is present in the parabigeminal nucleus (PBG), nucleus of the trapezoid body (NTB), and weakly expressed in the dorsal raphe nuclei (RN) and Pedunculopontine nuclei (PPN). (L) In the brainstem, *Cd24* expression was observed in the cerebellar nuclei (CBN), medial vestibular nucleus (MV), facial motor nucleus (VII), and ventral cochlear nucleus (VCO). The scale bar in panel L represents 5mm in panel E,1mm in panels A, C and K, 800μm in panel L, 500μm in panels D, F, G, I and J, and 100μm in panels B and H.
(TIF)

S2 Fig Immunohistochemistry of CD24 expression on an 8-week old adult mouse brain. (A) Strong CD24 expression in the olfactory bulb (especially the glomerular layer and rostral migratory stream). (B) In the entire lining of the ventricular zone at the level of the striatum, CD24 is present. (C) CD24 protein is observed the Reticular nucleus of the thalamus (RT). (D) At the level of the thalamus, strong CD24 expression is seen in the amygdala and the lateral posterior thalamic nucleus (mediocaudal part; LPMC). (E) The LPMC expression is maintained into the midbrain, where there is also very strong staining in the zonal layer and weaker expression in the ventral layers of the superior colliculus (SC). (F) The SC expression of CD24 is maintained throughout the midbrain. There is also CD24 staining surrounding the medial geniculate nucleus (MG). (G) Robust expression in the red nucleus. (H) In the hindbrain, there is strong expression in the nucleus of lateral lemniscus (NLL) and the superior olivary complex (SOC). (I-L) CD24 expression on sections of brain from the *Cd24-/-* mouse, demonstrating a lack of protein labelling at all levels of the CNS. Numbers on the periphery represent the plane of the coronal section relative to bregma. Scale bar in panel D represents 3mm in images A-D and I-L, and 1.5mm for images E-H.
(TIF)

S3 Fig Immunohistochemistry of TH expression in the *Cd24-/-* mouse. The DA system of the 2-year old *Cd24-/-* mouse (left column) differs little from that of the age-matched *Cd24+/+* mouse (right) across all of the coronal planes examined. Numbers on the periphery represent the plane of the coronal section relative to bregma. Scale bar represents 3mm.
(TIF)

S4 Fig Immunohistochemistry demonstrating the lack of GDNF neuroprotection at the level of the striatal neurites following 6-OHDA lesion. (A) GDNF expression in the striatum of a *Cd24-/-* mouse one-month post-AAV-GDNF striatal delivery. (B) AAV-GDNF transfection of the striatum resulted in an increase in CD24 expression in the SNpc on the injected side of the brain in *Cd24+/+* mouse, but not *Cd24-/-* mice (B'). (C-F) Three weeks post-6-OHDA delivery to the striatum, both groups of AAV-GDNF mice (both *Cd24+/+* and *Cd24-/-*) presented varying levels of TH+ fibre loss in the striatum. (C-C'' and D-D'' represent the worst cases in the *Cd24+/+* and *Cd24-/-* groups, respectively; while E-E'' and F-F'' represent the best cases in the *Cd24+/+* and *Cd24-/-* groups, respectively. Numbers on the periphery represent the plane of the coronal section relative to bregma. Scale bar in panel A represents 2mm in panel A,500μm in panels B-B', and 1mm in panels C-F''.
(TIF)

S5 Fig Fibre density analysis and stereological estimations at 21 days post 6-OHDA lesion compared to 12 and 70 day timepoints. (A) TH+ fibre density analysis of the striatum at each of the different time points used in this study, presented as percentage of the unlesioned intact striatum. (B) A representative example of the lesioned striatum of a WT mice at 21 days post 6-OHDA lesion. (C) Stereological estimations of TH+ cells in the SN of WT mice at each of the different time points used in this study, presented as percentage of the unlesioned intact SN. (D) Representative sections of midbrain at the level of fasciculus retroflexus (FR) and the medial lemniscus (ML) are presented. Numbers in the bottom right corner of panels B&D are co-ordinates representing the planes of the coronal section relative to bregma. Scale bar in panel D represents 2mm in panel B, and 500μm in panel D.
(TIF)

## Abbreviations

3V: Third ventricle
6-OHDA: 6-Hydroxydopamine
Amg: Amgydala nucleus
AP: Area postrema
CA1-4: Cornus ammonis region of hippocampus proper 1–4
CBN: Cerebellar nuclei
CD24: Cluster of differentiation 24
DAT: Dopamine Transporter
DEPC: Diethyl pryrocarbonate
DG: Dentate gyrus
DmnV: Dorsal motor nucleus of the vagus nerve
EAE: Experimental autoimmune encephalomyelitis
FR: Fasciculus retroflexus
GDNF: Glial cell-line derived neurotrophic factor
GPI: Glycosyl phosphatidylinositol
HSA: Heat-stable antigen
LC: Locus coeruleus
LPMC: Lateral posterior thalamic nucleus
Ly-52: Lymphocyte antigen 52
MG: Medial geniculate
ML: Medial lemniscus
MM: Mammillary bodies
Mtn: Medial terminal nucleus of the accessory optic tract
MV: Medial vestibular nucleus
O: Olivary nucleus
NLL: Nucleus of lateral lemniscus
NTB: Nucleus of the trapezoid body
P: Pyramid (of the medulla oblongata)
PBS: Phosphate buffered saline
PD: Parkinson’s disease
PBG: Parabigeminal nucleus
PBl: Parabrachial nucleus
PPN: Pedunculopontine nuclei
R: Red nucleus
RRF: Retrorubral field
RMS: Rostral Migratory Stream
RN: Raphe nucleus
S: Solitary nucleus
SC: Superior colliculus
SCN: Suprachiasmatic nucleus
SOC: Superior olivary complex
SNpc: Substantia nigra pars compacta
SNpr: Substantia nigra pars reticulata
Str: Striatum
SVZ: Subventricular zone
VII: Facial motor nucleus
VCO: Ventral cochlear nucleus
VTA: Ventral tegmental area
V: Motor nucleus of trigeminal
VZ: Ventricular zone
XII: Hypoglossal nucleus

## References

[1] Jankovic J. Parkinson’s disease: clinical features and diagnosis. J Neurol Neurosurg Psychiatry. 2008;79: 368–376. 10.1136/jnnp.2007.131045 18344392

[2] Damier P, Hirsch EC, Agid Y, Graybiel AM. The substantia nigra of the human brain. II. Patterns of loss of dopamine-containing neurons in Parkinson’s disease. Brain J Neurol. 1999;122 (Pt 8: 1437–48. 10.1093/brain/122.8.1437 10430830

[3] Javoy-Agid F, Agid Y. Is the mesocortical dopaminergic system involved in Parkinson disease? Neurology. 1980;30: 1326–1330. 6109265 10.1212/wnl.30.12.1326

[4] McRitchie DA, Cartwright HR, Halliday GM. Specific A10 dopaminergic nuclei in the midbrain degenerate in Parkinson’s disease. Exp Neurol. 1997;144: 202–213. 10.1006/exnr.1997.6418 9126172

[5] Del Tredici K, Braak H. Lewy pathology and neurodegeneration in premotor Parkinson’s disease. Mov Disord Off J Mov Disord Soc. 2012;27: 597–607. 10.1002/mds.24921 22508278

[6] Fang X, Zheng P, Tang J, Liu Y. CD24: from A to Z. Cell Mol Immunol. 2010;7: 100–103. 10.1038/cmi.2009.119 20154703 PMC4001892

[7] Liu Y, Jones B, Brady W, Janeway CA, Linsley PS, Linley PS. Co-stimulation of murine CD4 T cell growth: cooperation between B7 and heat-stable antigen. Eur J Immunol. 1992;22: 2855–2859. 10.1002/eji.1830221115 1385153

[8] Liu Y, Wenger RH, Zhao M, Nielsen PJ. Distinct costimulatory molecules are required for the induction of effector and memory cytotoxic T lymphocytes. J Exp Med. 1997;185: 251–262. 9016874 10.1084/jem.185.2.251 PMC2196124

[9] Wu Y, Zhou Q, Zheng P, Liu Y. CD28-independent induction of T helper cells and immunoglobulin class switches requires costimulation by the heat-stable antigen. J Exp Med. 1998;187: 1151–1156. 9529332 10.1084/jem.187.7.1151 PMC2212205

[10] Bai X-F, Li O, Zhou Q, Zhang H, Joshi PS, Zheng X, et al CD24 controls expansion and persistence of autoreactive T cells in the central nervous system during experimental autoimmune encephalomyelitis. J Exp Med. 2004;200: 447–58. 10.1084/jem.20040131 15314074 PMC2211938

[11] Liu J-Q, Carl JW, Joshi PS, RayChaudhury A, Pu X-A, Shi F-D, et al CD24 on the resident cells of the central nervous system enhances experimental autoimmune encephalomyelitis. J Immunol Baltim Md 1950. 2007;178: 6227–35. 10.4049/jimmunol.178.10.6227 17475850

[12] Chen G-Y, Tang J, Zheng P, Liu Y. CD24 and Siglec-10 selectively repress tissue damage-induced immune responses. Science. 2009;323: 1722–1725. 10.1126/science.1168988 19264983 PMC2765686

[13] Gao L, Hidalgo-Figueroa M, Escudero LM, Díaz-Martín J, López-Barneo J, Pascual A. Age-mediated transcriptomic changes in adult mouse substantia nigra. PloS One. 2013;8: e62456 10.1371/journal.pone.0062456 23638090 PMC3640071

[14] Chung CY, Seo H, Sonntag KC, Brooks A, Lin L, Isacson O. Cell type-specific gene expression of midbrain dopaminergic neurons reveals molecules involved in their vulnerability and protection. Hum Mol Genet. 2005;14: 1709–25. 10.1093/hmg/ddi178 15888489 PMC2674782

[15] Brichta L, Shin W, Jackson-Lewis V, Blesa J, Yap E-L, Walker Z, et al Identification of neurodegenerative factors using translatome-regulatory network analysis. Nat Neurosci. 2015;18: 1325–1333. 10.1038/nn.4070 26214373 PMC4763340

[16] Lewandowski NM, Ju S, Verbitsky M, Ross B, Geddie ML, Rockenstein E, et al Polyamine pathway contributes to the pathogenesis of Parkinson disease. Proc Natl Acad Sci U S A. 2010;107: 16970–16975. 10.1073/pnas.1011751107 20837543 PMC2947879

[17] McClung CA, Nestler EJ, Zachariou V. Regulation of gene expression by chronic morphine and morphine withdrawal in the locus ceruleus and ventral tegmental area. J Neurosci Off J Soc Neurosci. 2005;25: 6005–6015. 10.1523/JNEUROSCI.0062-05.2005 PMC6724795 15976090

[18] Nielsen PJ, Lorenz B, Müller AM, Wenger RH, Brombacher F, Simon M, et al Altered erythrocytes and a leaky block in B-cell development in CD24/HSA-deficient mice. Blood. 1997;89: 1058–67. 9028339

[19] Breysse N, Carlsson T, Winkler C, Björklund A, Kirik D. The functional impact of the intrastriatal dopamine neuron grafts in parkinsonian rats is reduced with advancing disease. J Neurosci Off J Soc Neurosci. 2007;27: 5849–5856. 10.1523/JNEUROSCI.0626-07.2007 PMC6672262 17537955

[20] Kirik D, Winkler C, Björklund A. Growth and functional efficacy of intrastriatal nigral transplants depend on the extent of nigrostriatal degeneration. J Neurosci Off J Soc Neurosci. 2001;21: 2889–2896. 10.1523/JNEUROSCI.21-08-02889.2001 PMC6762534 11306640

[21] de Chaumont F, Coura RD-S, Serreau P, Cressant A, Chabout J, Granon S, et al Computerized video analysis of social interactions in mice. Nat Methods. 2012;9: 410–417. 10.1038/nmeth.1924 22388289

[22] de Chaumont F, Dallongeville S, Chenouard N, Hervé N, Pop S, Provoost T, et al Icy: an open bioimage informatics platform for extended reproducible research. Nat Methods. 2012;9: 690–696. 10.1038/nmeth.2075 22743774

[23] Stott SRW, Barker RA. Time course of dopamine neuron loss and glial response in the 6-OHDA striatal mouse model of Parkinson’s disease. Eur J Neurosci. 2014;39: 1042–1056. 10.1111/ejn.12459 24372914

[24] Guillery RW, Herrup K. Quantification without pontification: choosing a method for counting objects in sectioned tissues. J Comp Neurol. 1997;386: 2–7. 9303520 10.1002/(sici)1096-9861(19970915)386:1<2::aid-cne2>3.0.co;2-6

[25] West MJ. Stereological methods for estimating the total number of neurons and synapses: issues of precision and bias. Trends Neurosci. 1999;22: 51–61. 10092043 10.1016/s0166-2236(98)01362-9

[26] Carlsson T, Carta M, Winkler C, Björklund A, Kirik D. Serotonin neuron transplants exacerbate L-DOPA-induced dyskinesias in a rat model of Parkinson’s disease. J Neurosci Off J Soc Neurosci. 2007;27: 8011–8022. 10.1523/JNEUROSCI.2079-07.2007 PMC6672736 17652591

[27] Decressac M, Mattsson B, Lundblad M, Weikop P, Björklund A. Progressive neurodegenerative and behavioural changes induced by AAV-mediated overexpression of α-synuclein in midbrain dopamine neurons. Neurobiol Dis. 2012;45: 939–953. 22182688 10.1016/j.nbd.2011.12.013

[28] Braak H, Del Tredici K, Rüb U, De Vos RAI, Jansen Steur ENH, Braak E. Staging of brain pathology related to sporadic Parkinson’s disease. Neurobiol Aging. 2003;24: 197–211. 12498954 10.1016/s0197-4580(02)00065-9

[29] Daniel SE, Hawkes CH. Preliminary diagnosis of Parkinson’s disease by olfactory bulb pathology. Lancet Lond Engl. 1992;340: 186. 10.1016/0140-6736(92)93275-r 1352606

[30] Braak H, Braak E, Yilmazer D, de Vos RA, Jansen EN, Bohl J, et al Amygdala pathology in Parkinson’s disease. Acta Neuropathol (Berl). 1994;88: 493–500. 7879596 10.1007/BF00296485

[31] Goto S, Hirano A. Catecholaminergic neurons in the parabrachial nucleus of normal individuals and patients with idiopathic Parkinson’s disease. Ann Neurol. 1991;30: 192–196. 10.1002/ana.410300211 1680303

[32] Jellinger K. The pedunculopontine nucleus in Parkinson’s disease, progressive supranuclear palsy and Alzheimer’s disease. J Neurol Neurosurg Psychiatry. 1988;51: 540–543. 3379428 10.1136/jnnp.51.4.540 PMC1032970

[33] Calaora V, Chazal G, Nielsen PJ, Rougon G, Moreau H. mCD24 expression in the developing mouse brain and in zones of secondary neurogenesis in the adult. Neuroscience. 1996;73: 581–94. 8783272 10.1016/0306-4522(96)00042-5

[34] Jacobs FMJ, Smits SM, Noorlander CW, von Oerthel L, van der Linden AJA, Burbach JPH, et al Retinoic acid counteracts developmental defects in the substantia nigra caused by Pitx3 deficiency. Dev Camb Engl. 2007;134: 2673–2684. 10.1242/dev.02865 17592014

[35] Mendez I, Sanchez-Pernaute R, Cooper O, Viñuela A, Ferrari D, Björklund L, et al Cell type analysis of functional fetal dopamine cell suspension transplants in the striatum and substantia nigra of patients with Parkinson’s disease. Brain J Neurol. 2005;128: 1498–1510. 10.1093/brain/awh510 PMC2610438 15872020

[36] Thompson L, Barraud P, Andersson E, Kirik D, Björklund A. Identification of dopaminergic neurons of nigral and ventral tegmental area subtypes in grafts of fetal ventral mesencephalon based on cell morphology, protein expression, and efferent projections. J Neurosci Off J Soc Neurosci. 2005;25: 6467–6477. 10.1523/JNEUROSCI.1676-05.2005 PMC6725273 16000637

[37] Jevsek M, Jaworski A, Polo-Parada L, Kim N, Fan J, Landmesser LT, et al CD24 is expressed by myofiber synaptic nuclei and regulates synaptic transmission. Proc Natl Acad Sci U S A. 2006;103: 6374–9. 10.1073/pnas.0601468103 16606832 PMC1435367

[38] Engler H, Doenlen R, Riether C, Engler A, Niemi M-B, Besedovsky HO, et al Time-dependent alterations of peripheral immune parameters after nigrostriatal dopamine depletion in a rat model of Parkinson’s disease. Brain Behav Immun. 2009;23: 518–526. 19486644 10.1016/j.bbi.2009.01.018

[39] Brochard V, Combadière B, Prigent A, Laouar Y, Perrin A, Beray-Berthat V, et al Infiltration of CD4+ lymphocytes into the brain contributes to neurodegeneration in a mouse model of Parkinson disease. J Clin Invest. 2009;119: 182–192. 10.1172/JCI36470 19104149 PMC2613467

[40] Bai XF, Liu JQ, Liu X, Guo Y, Cox K, Wen J, et al The heat-stable antigen determines pathogenicity of self-reactive T cells in experimental autoimmune encephalomyelitis. J Clin Invest. 2000;105: 1227–1232. 10.1172/JCI9012 10791997 PMC315444

[41] Lundblad M, Picconi B, Lindgren H, Cenci MA. A model of L-DOPA-induced dyskinesia in 6-hydroxydopamine lesioned mice: relation to motor and cellular parameters of nigrostriatal function. Neurobiol Dis. 2004;16: 110–123. 10.1016/j.nbd.2004.01.007 15207268

[42] Jeon BS, Jackson-Lewis V, Burke RE. 6-Hydroxydopamine lesion of the rat substantia nigra: time course and morphology of cell death. Neurodegener J Neurodegener Disord Neuroprotection Neuroregeneration. 1995;4: 131–137. 10.1006/neur.1995.0016 7583676

[43] Alvarez-Fischer D, Henze C, Strenzke C, Westrich J, Ferger B, Höglinger GU, et al Characterization of the striatal 6-OHDA model of Parkinson’s disease in wild type and alpha-synuclein-deleted mice. Exp Neurol. 2008;210: 182–193. 10.1016/j.expneurol.2007.10.012 18053987

[44] Christophersen NS, Grønborg M, Petersen TN, Fjord-Larsen L, Jørgensen JR, Juliusson B, et al Midbrain expression of Delta-like 1 homologue is regulated by GDNF and is associated with dopaminergic differentiation. Exp Neurol. 2007;204: 791–801. 10.1016/j.expneurol.2007.01.014 17320866

[45] Manfredsson FP, Tumer N, Erdos B, Landa T, Broxson CS, Sullivan LF, et al Nigrostriatal rAAV-mediated GDNF overexpression induces robust weight loss in a rat model of age-related obesity. Mol Ther J Am Soc Gene Ther. Nature Publishing Group; 2009;17: 980–91. 10.1038/mt.2009.45 PMC2835185 19277011

[46] Stott SRW, Metzakopian E, Lin W, Kaestner KH, Hen R, Ang S-L. Foxa1 and foxa2 are required for the maintenance of dopaminergic properties in ventral midbrain neurons at late embryonic stages. J Neurosci Off J Soc Neurosci. 2013;33: 8022–8034. 10.1523/JNEUROSCI.4774-12.2013 PMC6618950 23637192

[47] Reyes S, Fu Y, Double KL, Cottam V, Thompson LH, Kirik D, et al Trophic factors differentiate dopamine neurons vulnerable to Parkinson’s disease. Neurobiol Aging. 2013;34: 873–886. 10.1016/j.neurobiolaging.2012.07.019 22926168

[48] Shewan D, Calaora V, Nielsen P, Cohen J, Rougon G, Moreau H. mCD24, a glycoprotein transiently expressed by neurons, is an inhibitor of neurite outgrowth. J Neurosci Off J Soc Neurosci. 1996;16: 2624–2634. 10.1523/JNEUROSCI.16-08-02624.1996 PMC6578769 8786438

[49] Kleene R, Yang H, Kutsche M, Schachner M. The neural recognition molecule L1 is a sialic acid-binding lectin for CD24, which induces promotion and inhibition of neurite outgrowth. J Biol Chem. 2001;276: 21656–21663. 10.1074/jbc.M101790200 11283023

[50] Pandey V, Jung Y, Kang J, Steiner M, Qian P-X, Banerjee A, et al Artemin Reduces Sensitivity to Doxorubicin and Paclitaxel in Endometrial Carcinoma Cells through Specific Regulation of CD24. Transl Oncol. 2010;3: 218–29. 20689763 10.1593/tlo.09325 PMC2915413

[51] Schäck LM, Buettner M, Wirth A, Neunaber C, Krettek C, Hoffmann A, et al Expression of CD24 in Human Bone Marrow-Derived Mesenchymal Stromal Cells Is Regulated by TGFβ3 and Induces a Myofibroblast-Like Genotype. Stem Cells Int. 2016;2016: 1319578 10.1155/2016/1319578 26788063 PMC4691640

[52] Kollaee A, Ghaffarpor M, Pourmahmoudian H, Shahbazi M, Zamani M. Investigation of CD24 and its expression in Iranian relapsing-remitting multiple sclerosis. Int J Neurosci. 2011;121: 684–690. 10.3109/00207454.2011.610529 21815873

[53] Zhou Q, Rammohan K, Lin S, Robinson N, Li O, Liu X, et al CD24 is a genetic modifier for risk and progression of multiple sclerosis. Proc Natl Acad Sci U S A. 2003;100: 15041–15046. 10.1073/pnas.2533866100 14657362 PMC299898

[54] Diaz-Gallo L-M, Medrano LM, Gómez-García M, Cardeña C, Rodrigo L, Mendoza JL, et al Analysis of the influence of two CD24 genetic variants in Crohn’s disease and ulcerative colitis. Hum Immunol. 2011;72: 969–972. 21684315 10.1016/j.humimm.2011.05.028

[55] Sánchez E, Abelson A-K, Sabio JM, González-Gay MA, Ortego-Centeno N, Jiménez-Alonso J, et al Association of a CD24 gene polymorphism with susceptibility to systemic lupus erythematosus. Arthritis Rheum. 2007;56: 3080–3086. 10.1002/art.22871 17763438

[56] Decressac M, Kadkhodaei B, Mattsson B, Laguna A, Perlmann T, Björklund A. α-Synuclein-induced down-regulation of Nurr1 disrupts GDNF signaling in nigral dopamine neurons. Sci Transl Med. 2012;4: 163ra156 10.1126/scitranslmed.3004676 23220632

