# Supplementary figures and images for: CD24 expression does not affect dopamine neuronal survival in a mouse model of Parkinson's disease
Source: PLoS One. 2017 Feb 9;12(2):e0171748. doi: 10.1371/journal.pone.0171748 (PMC5300212; doi:10.1371/journal.pone.0171748)

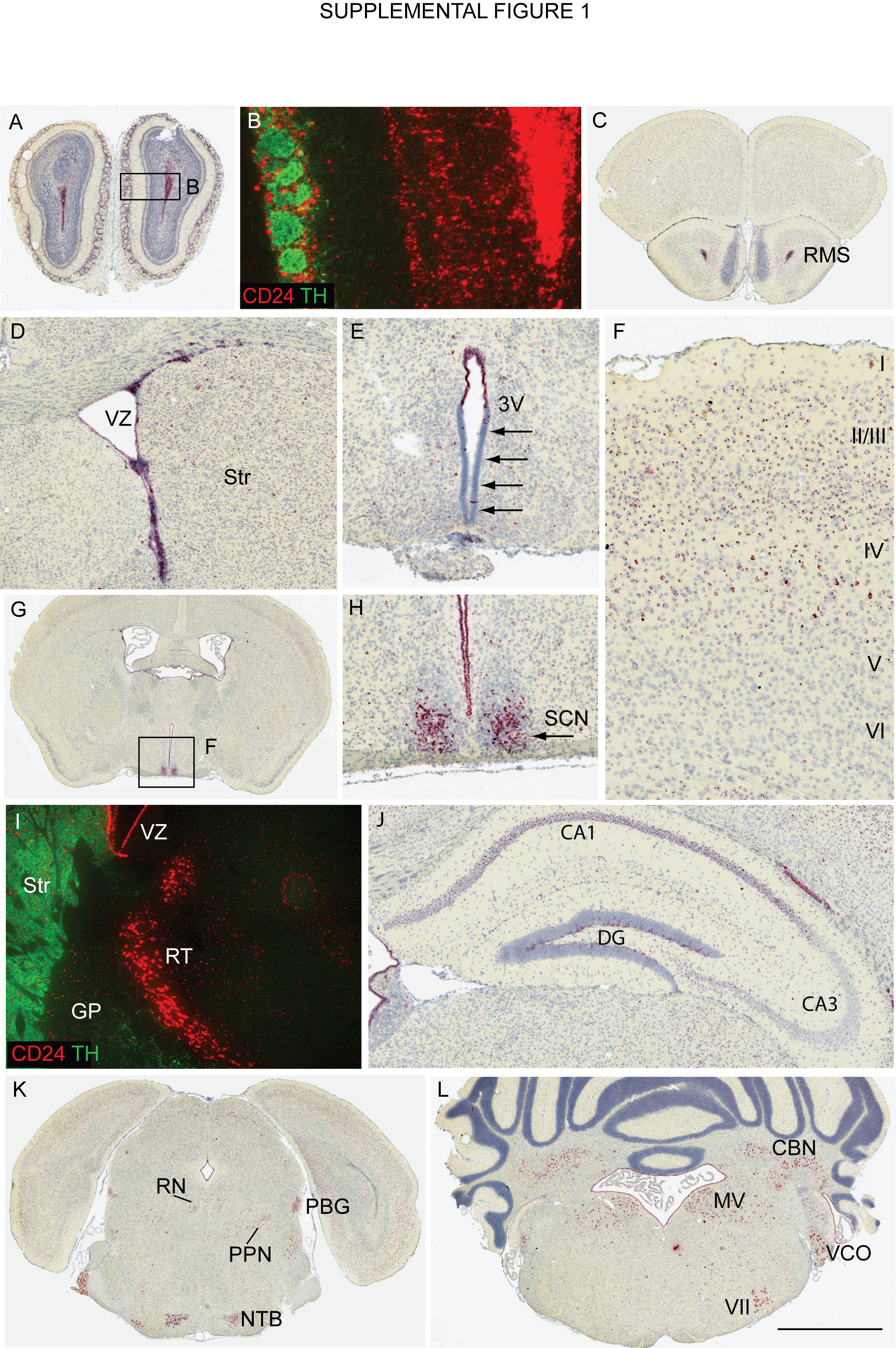

Supplement: S1 Fig — (A) Cd24 transcript (red; with haematoxylin counterstain) expression is present in the olfactory bulbs. (B) A high magnification image of the dashed boxed region in panel A, providing Cd24 expression overlapped with TH immunofluorescent staining in the olfactory bulb. (C) Robust Cd24 transcripts in the rostral migratory stream (RMS) and 'salt and pepper' distribution throughout the frontal cortex. (D) Strong expression of Cd24 in the lining of the ventricular zone (VZ) across all levels of the striatum (Str). (E) Cd24 expression at the level of the thalamus. (F) The dashed-boxed area of panel E, providing a high magnification image of Cd24 expression in the suprachiasmatic nucleus (SCN). (G) An image of the third ventricle (3V) at the level of the caudal hypothalamus; arrows indicating the area of no Cd24 expression (a consistent feature across 3 brains analysed). (H) Cd24 expression in the cortex, layers are indicated on the right-hand side. (I) Cd24 expression in the hippocampus, especially within the dentate gyrus (DG) and CA1, but not CA3. (J) Immunofluorescent staining of TH expression in the Striatum (Str) and the neighbouring globus pallidus (GP), in combination with Cd24 expression in the Reticular nucleus of the thalamus (RT) and lining of the VZ. (K) In the hindbrain, Cd24 is present in the parabigeminal nucleus (PBG), nucleus of the trapezoid body (NTB), and weakly expressed in the dorsal raphe nuclei (RN) and Pedunculopontine nuclei (PPN). (L) In the brainstem, Cd24 expression was observed in the cerebellar nuclei (CBN), medial vestibular nucleus (MV), facial motor nucleus (VII), and ventral cochlear nucleus (VCO). The scale bar in panel L represents 5mm in panel E,1mm in panels A, C and K, 800μm in panel L, 500μm in panels D, F, G, I and J, and 100μm in panels B and H. (TIF) [file pone.0171748.s001.tif]

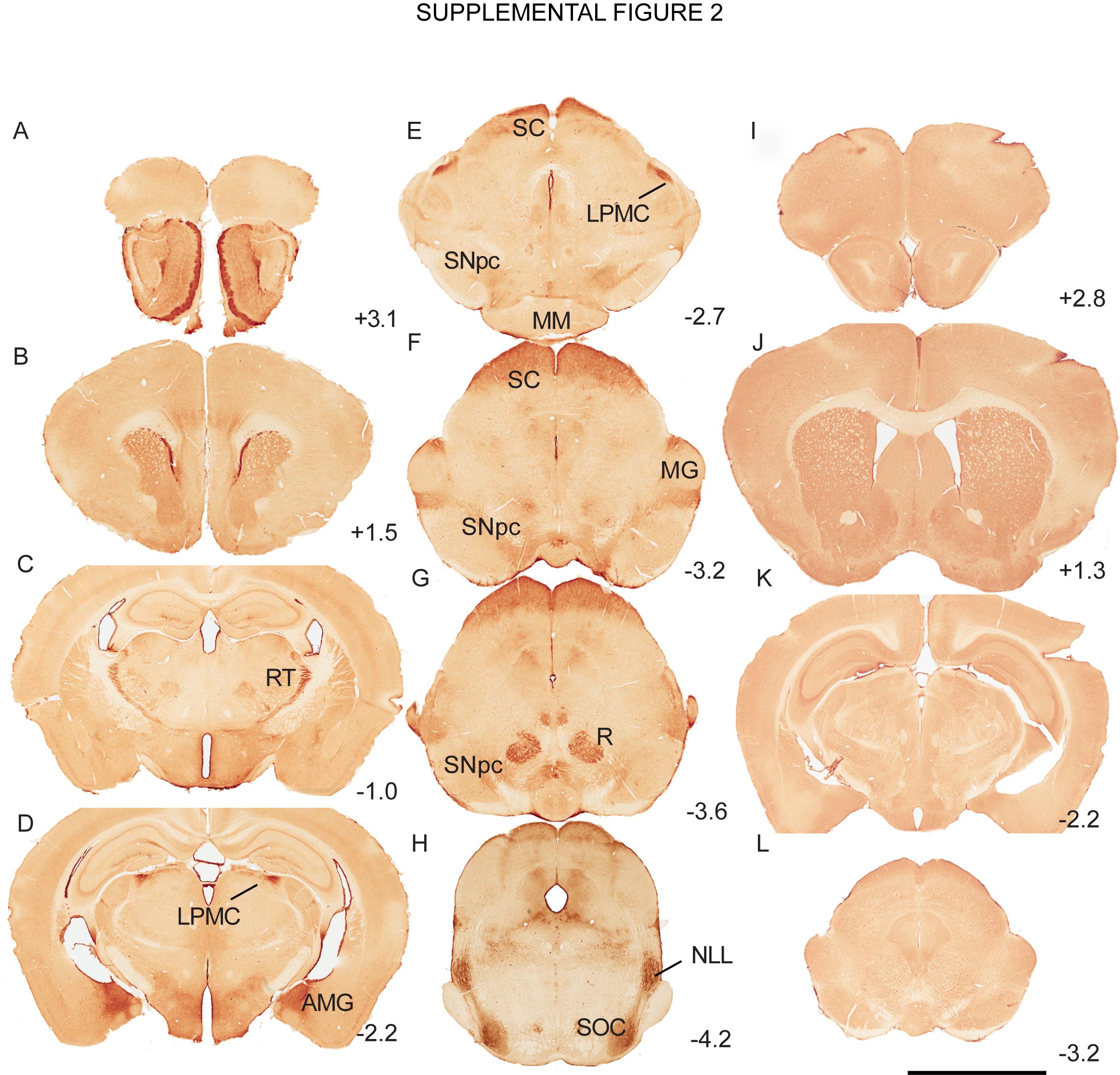

Supplement: S2 Fig — (A) Strong CD24 expression in the olfactory bulb (especially the glomerular layer and rostral migratory stream). (B) In the entire lining of the ventricular zone at the level of the striatum, CD24 is present. (C) CD24 protein is observed the Reticular nucleus of the thalamus (RT). (D) At the level of the thalamus, strong CD24 expression is seen in the amygdala and the lateral posterior thalamic nucleus (mediocaudal part; LPMC). (E) The LPMC expression is maintained into the midbrain, where there is also very strong staining in the zonal layer and weaker expression in the ventral layers of the superior colliculus (SC). (F) The SC expression of CD24 is maintained throughout the midbrain. There is also CD24 staining surrounding the medial geniculate nucleus (MG). (G) Robust expression in the red nucleus. (H) In the hindbrain, there is strong expression in the nucleus of lateral lemniscus (NLL) and the superior olivary complex (SOC). (I-L) CD24 expression on sections of brain from the Cd24-/- mouse, demonstrating a lack of protein labelling at all levels of the CNS. Numbers on the periphery represent the plane of the coronal section relative to bregma. Scale bar in panel D represents 3mm in images A-D and I-L, and 1.5mm for images E-H. (TIF) [file pone.0171748.s002.tif]

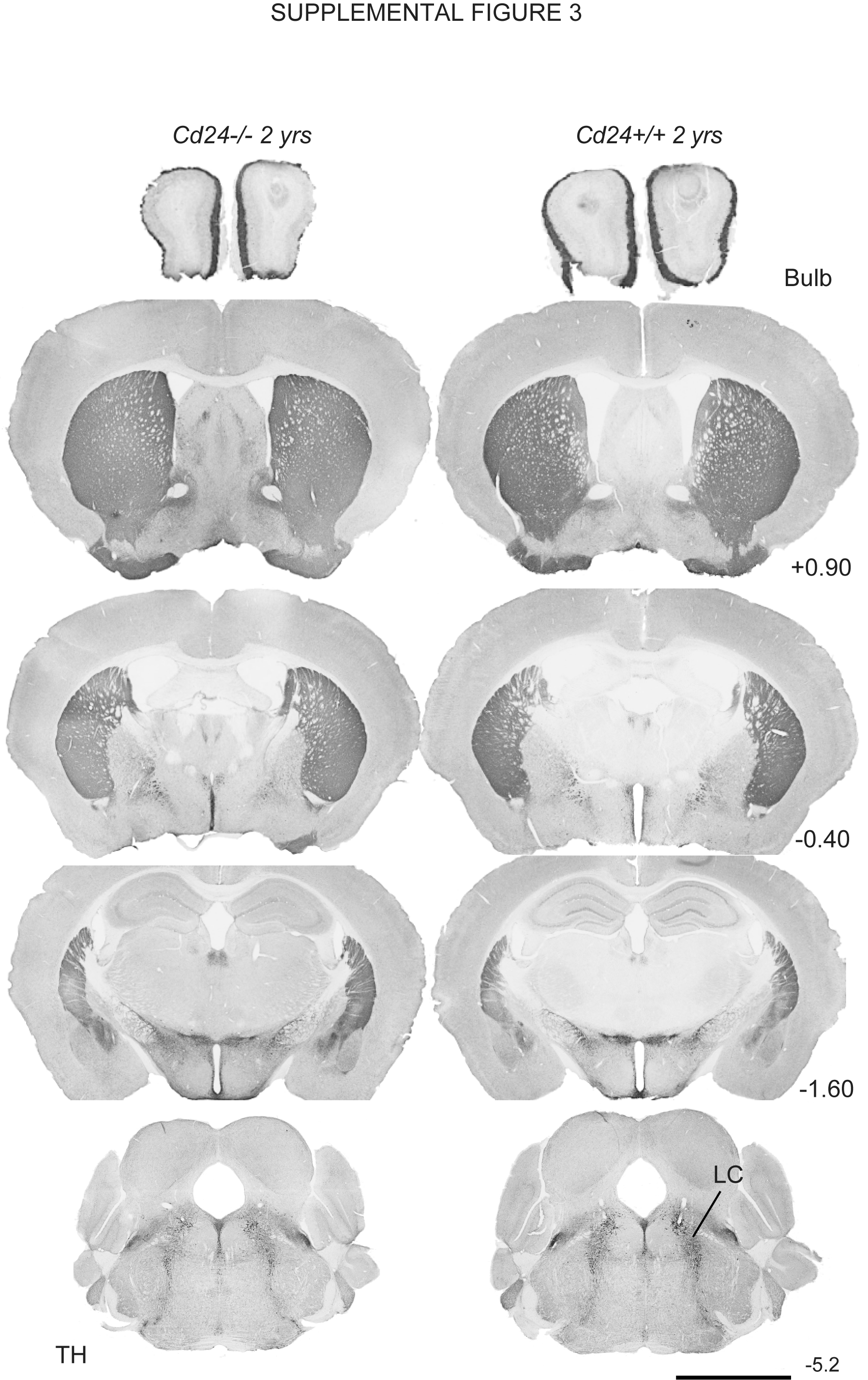

Supplement: S3 Fig — The DA system of the 2-year old Cd24-/- mouse (left column) differs little from that of the age-matched Cd24+/+ mouse (right) across all of the coronal planes examined. Numbers on the periphery represent the plane of the coronal section relative to bregma. Scale bar represents 3mm. (TIF) [file pone.0171748.s003.tif]

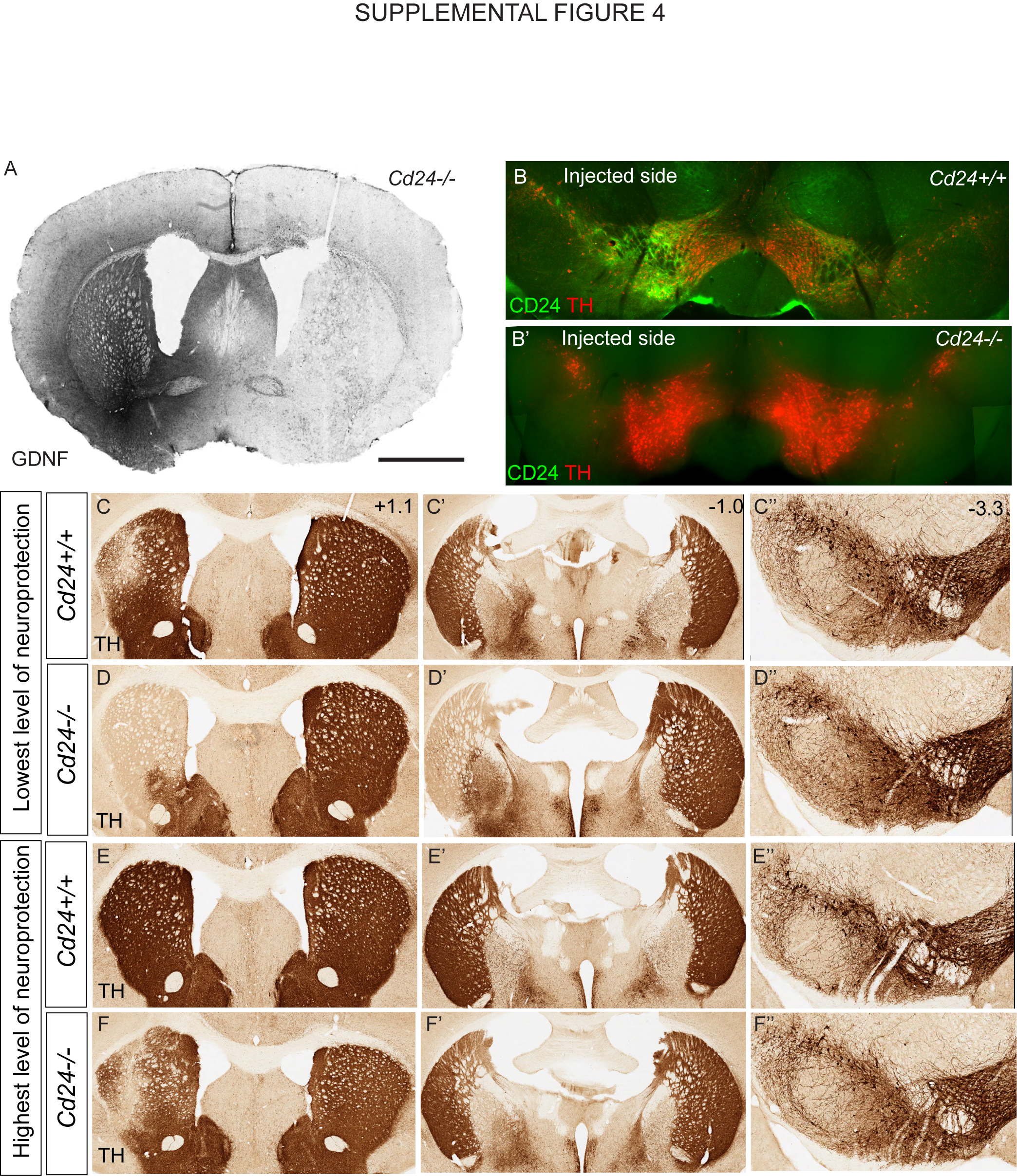

Supplement: S4 Fig — (A) GDNF expression in the striatum of a Cd24-/- mouse one-month post-AAV-GDNF striatal delivery. (B) AAV-GDNF transfection of the striatum resulted in an increase in CD24 expression in the SNpc on the injected side of the brain in Cd24+/+ mouse, but not Cd24-/- mice (B'). (C-F) Three weeks post-6-OHDA delivery to the striatum, both groups of AAV-GDNF mice (both Cd24+/+ and Cd24-/-) presented varying levels of TH+ fibre loss in the striatum. (C-C'' and D-D'' represent the worst cases in the Cd24+/+ and Cd24-/- groups, respectively; while E-E'' and F-F'' represent the best cases in the Cd24+/+ and Cd24-/- groups, respectively. Numbers on the periphery represent the plane of the coronal section relative to bregma. Scale bar in panel A represents 2mm in panel A,500μm in panels B-B', and 1mm in panels C-F''. (TIF) [file pone.0171748.s004.tif]

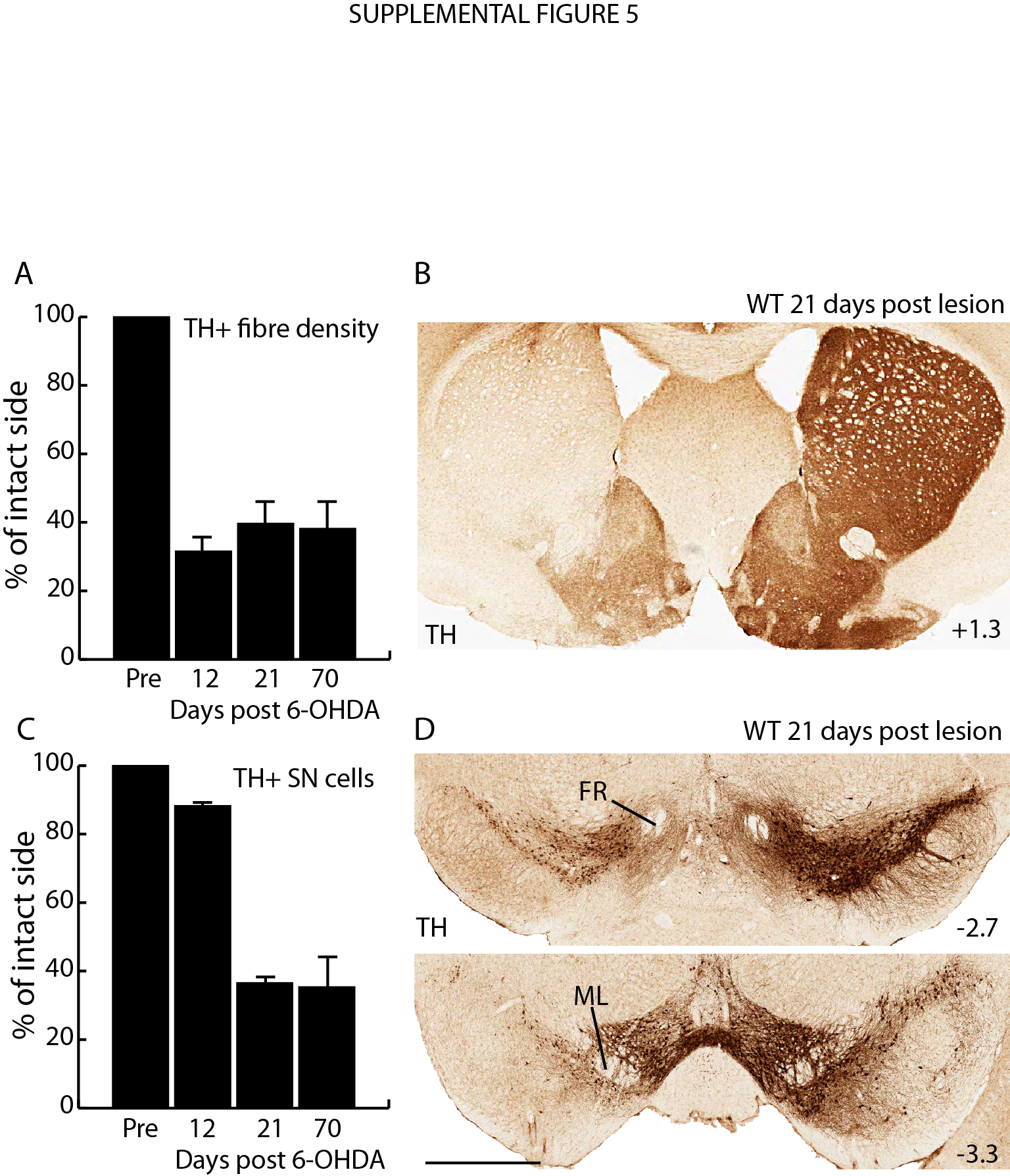

Supplement: S5 Fig — (A) TH+ fibre density analysis of the striatum at each of the different time points used in this study, presented as percentage of the unlesioned intact striatum. (B) A representative example of the lesioned striatum of a WT mice at 21 days post 6-OHDA lesion. (C) Stereological estimations of TH+ cells in the SN of WT mice at each of the different time points used in this study, presented as percentage of the unlesioned intact SN. (D) Representative sections of midbrain at the level of fasciculus retroflexus (FR) and the medial lemniscus (ML) are presented. Numbers in the bottom right corner of panels B&D are co-ordinates representing the planes of the coronal section relative to bregma. Scale bar in panel D represents 2mm in panel B, and 500μm in panel D. (TIF) [file pone.0171748.s005.tif]
